# Experience-driven meaning affects lexical choices during language production

**DOI:** 10.1177/17470218221125425

**Published:** 2022-10-06

**Authors:** Anne Vogt, Barbara Kaup, Rasha Abdel Rahman

**Affiliations:** 1 Department of Psychology, Humboldt-Universität zu Berlin, Berlin, Germany; 2 Berlin School of Mind and Brain, Berlin, Germany; 3 University of Tübingen, Tübingen, Germany

**Keywords:** Language production, experiential traces, language grounding, hybrid models, lexical selection, semantic processing

## Abstract

The role of meaning facets based on sensorimotor experiences is well investigated in comprehension but has received little attention in language production research. In two experiments, we investigated whether experiential traces of space influenced lexical choices when participants completed visually presented sentence fragments (e.g., “You are at the sea and you see a . . .”) with spoken nouns (e.g., “dolphin,” “palm tree”). The words were presented consecutively in an ascending or descending direction, starting from the centre of the screen. These physical spatial cues did not influence lexical choices. However, the produced nouns met the spatial characteristics of the broader sentence contexts such that the typical spatial locations of the produced noun referents were predicted by the location of the situations described by the sentence fragments (i.e., upper or lower sphere). By including distributional semantic similarity measures derived from computing cosine values between sentence nouns and produced nouns using a web-based text corpus, we show that the meaning dimension of “location in space” guides lexical selection during speaking. We discuss the relation of this spatial meaning dimension to accounts of experientially grounded and usage-based theories of language processing and their combination in hybrid approaches. In doing so, we contribute to a more comprehensive understanding of the many facets of meaning processing during language production and their impact on the words we select to express verbal messages.

A central process during language production is the selection of the right words to express an intended meaning. While the role of some meaning aspects—such as categorical relations—is well investigated, little is known about others (Abdel Rahman & Melinger, 2019). Specifically, and in contrast to language comprehension, little is known about meaning aspects grounded in sensorimotor experiences. This is surprising because we frequently talk about our sensations and experiences in everyday life. Therefore, meaning aspects linked to our sensory experiences seem fundamental in language production.

This study was designed to investigate the influences of experientially grounded meaning on lexical-semantic processing during language production. Furthermore, we relate sensorimotor experiences to a measure of semantic similarity using linguistic distributional measures of meaning relations.

## Semantic relations in language production

When speakers plan to produce a message, meaning representations at the conceptual level and word representations at the lexical level (lemmas)—and semantically related conceptual and lexical entries—are activated, and the target lemma is selected from among these co-activated alternatives (Caramazza, 1997; Dell, 1986; Levelt et al., 1999; Mahon et al., 2007; Oppenheim et al., 2010). Evidence of lexical-semantic factors influencing lexical selection stems from context effects induced by displaying constraining versus non-constraining sentences before asking individuals to name a picture (Hustá et al., 2021), from context effects by previously named related pictures (e.g., in the cyclic blocking and continuous naming paradigm; Belke et al., 2005; Howard et al., 2006), or simultaneously presented related distractor words (in the picture-word interference paradigm; e.g., Glaser & Düngelhoff, 1984; for a recent discussion, see Bürki et al., 2020; Roelofs, 2018). Typically, categorical semantic relations have been investigated in these paradigms. However, the meaning of verbal messages is multifaceted and may as well contain information about associations, part-whole relations, thematic links, and social and emotional meaning aspects. Therefore, it should not be reduced to categorical relations (Abdel Rahman & Melinger, 2019; Jackson et al., 2015), but investigations of non-categorical relations during lexical selection are comparatively rare and have focused on thematic, situational, or associative relations (Abdel Rahman & Melinger, 2007, 2019; Alario et al., 2000; Aristei & Abdel Rahman, 2013; Costa et al., 2005; Damian & Spalek, 2014; de Zubicaray et al., 2013; La Heij et al., 1990; Lin et al., 2021; Rose & Abdel Rahman, 2016). Crucially, lexical-semantic processing is not confined to traditionally investigated semantic relations and may include a much wider range of meaning facets based on sensory experiences, such as aspects of sound, shape, and colour which have been shown to play a role during language production (de Zubicaray et al., 2018; Mädebach et al., 2018; Redmann et al., 2014).

## Experientially grounded representations in language comprehension

Experiential grounding refers to the idea that the multimodal—and often bodily—experiences we have made leave experiential traces in our brain and become tied to our knowledge about these objects, situations, or actions (Barsalou, 2008) and, consequently, to the linguistic constructions and words used in those situations (e.g., Lynott et al., 2020; Zwaan & Madden, 2005). Due to its strong link to bodily sensations, this line of work is often referred to as embodiment or embodied cognition. We use the phrase “experiential grounding” throughout this article to highlight that not all experiences are based on bodily sensations. From this perspective, concepts can be understood as modality-specific, experience-dependent, and flexible representations in distributed neural networks which include, but are not restricted to, sensorimotor areas of the brain (Kiefer & Pulvermüller, 2012). Accessing these concepts as, for example, when retrieving word meanings involves a partial reactivation of the same brain processes that are active when experiencing the objects, situations, or actions to which these concepts refer. This is also referred to as experiential simulation (Barsalou, 1999; Pecher & Zwaan, 2005). These semantic effects occur within 100–200 ms after presentation of verbal stimuli, near simultaneously to a range of psycholinguistic processes during comprehension (Pulvermüller et al., 2009; García et al., 2019), and can therefore not be reduced to post-comprehension processes (Hoenig et al., 2008). Furthermore, sensorimotor activations are modulated by context, allowing for a high degree of flexibility and fluency in the language comprehension system (Aravena et al., 2014; Hoenig et al., 2008).

There is ample evidence that experientially grounded meaning plays an essential role in conceptual knowledge (e.g., Binder & Desai, 2011) and language comprehension (for overviews, see Bergen, 2015; Kaup et al., 2015; Meteyard et al., 2012; Pulvermüller, 2018).

## Language–space associations

A particularly well-investigated domain of experiential grounding in language comprehension is the association of language and space in the vertical dimension. Spatial locations do not by themselves form a natural category and there is no a priori thematic or associative link between objects sharing the same space within the upper or lower sphere (e.g., between “kite,” “bird’s nest,” and “crown” as objects typically found in the upper sphere of our world). Therefore, experiential traces of space seem particularly well suited to investigate the role of situational and experientially grounded meaning during language processing, as spatial locations can easily be inferred but are an implicit aspect of meaning. Due to the reactivations of actual experiences during concept acquisition, processing nouns referring to objects with a typical location in space leads to an orientation of attention towards this location (e.g., Dudschig et al., 2012; Estes et al., 2008; Öttl et al., 2017). These reactivations of experiential traces of space are tied to simulations of contexts or events in which an object typically appears and cannot be deduced to abstract meaning features, such as “up” or “down” (Ostarek & Vigliocco, 2017). Furthermore, spatial cues linked to situations can facilitate the accessibility of words, as has been shown in an anagram-solving task (Berndt et al., 2018). Most studies on language-space associations have focused on spatial compatibility effects where the dependent measure bears spatial characteristics, such as an upward or downward movement (Lachmair et al., 2011), thus investigating an effect of language on non-linguistic tasks. Furthermore, some studies used non-linguistic cues and investigated whether this influenced concurrent language processing. For example, Lachmair and colleagues (2016b) changed the body position of their participants between an upright or a head-down position. They found that participants remembered more up-words in the upright position and more down-words in the head-down position. In another study, vertical visual motion of dots on a screen had an impact on a lexical decision task when participants were presented with verbs denoting upward or downward movement, such as “rise” or “fall” (Dudschig et al., 2013; Meteyard et al., 2008). Thus, perception of motion can influence language comprehension (see also Kaschak et al., 2005), hinting at a link between visual and semantic processes.

## Experiential grounding and language production

While experiential grounding in comprehension is well investigated (see above), comparatively little is known about the potential role of experientially grounded meaning in language production, and it is unclear whether experiential traces are among the meaning factors that determine which lexical candidates are selected for articulation.

Two of the few studies suggesting that experientially grounded motor information may influence subsequent language production used a cyclic naming paradigm. In this paradigm, visually depicted actions were blocked according to their effector (hands/arms vs feet) and an interference effect for naming action verbs was found (de Zubicaray et al., 2017; Hirschfeld & Zwitserlood, 2012). In a second experiment by Hirschfeld and Zwitserlood (2012), participants were asked to produce action verbs for depicted actions while executing a concurrent motor task. When the effector of a depicted action (e.g., foot for the activity of jumping) matched the effector which had to be used for the concurrent motor task, interference in naming was observed, too. However, according to Hirschfeld and Zwitserlood, the results are also compatible with the view, that abstract foot- or hand-related semantic features were co-activated by the movements, spreading to abstract effector-related concepts, such as, for example, “part of the lower extremities,” “has toes/fingers,” and “used for walking/manipulating objects,” which then lead to competition between activated lexical nodes (see also Vigliocco et al., 2002). Therefore, they argue that their findings might not be interpreted as clear-cut evidence for a direct functional role of experientially grounded conceptual representations in language production.

Similar results have been obtained in other picture naming tasks. Investigating the motor domain, Witt et al. (2010) asked their participants to squeeze a ball in one hand, slowing down the naming of tools whose handles faced the squeezing hand compared with naming animals (but see Matheson et al., 2014). In an object naming task which was combined with a concurrent manual task, an increase in object naming errors was found which was related to the degree of experience subjects had in touching the depicted objects: for frequently manipulated objects, naming was more difficult when the concurrent motion task engaged the hands in a way which would make interaction with the real object impossible (Yee et al., 2013; for similar results using rTMS, see Pobric et al., 2010).

Furthermore, patients with motion-related neurological diseases, such as Parkinson’s, show increased difficulties in verb-naming tasks as the degree of motor content of the depicted actions increases (Herrera et al., 2012).

Asking participants to provide a verbal label for a given definition, Fargier et al. (2019) found that words which are strongly grounded in sensorimotor and emotional experiences are retrieved faster than words which are grounded to a lesser degree, irrespective of their concreteness. These results seem to support the importance of experientially grounded meaning aspects for lexical retrieval.

Taken together, few studies have investigated experientially grounded meaning in language production. Among those, some have investigated the role of experiential meaning in conceptual representations in general, employing mainly naming tasks, but without directly focusing on language production (Matheson et al., 2014; Mulatti et al., 2014; Sixtus et al., 2018; Witt et al., 2010; Yee et al., 2013). Furthermore, other studies provide little or inconsistent evidence concerning the role of experiential traces in lexical selection. First, it is still unclear whether the involvement of sensorimotor simulations during picture naming is necessary (de Zubicaray et al., 2017; Hirschfeld & Zwitserlood, 2012). Second, the activation of sensorimotor traces seems to be highly context-specific (Ben-Haim et al., 2015; Matheson et al., 2014). Moreover, there is evidence for both facilitation and interference of lexical access when providing information which boosts experiential simulations (de Zubicaray et al., 2017; Hirschfeld & Zwitserlood, 2012; Mulatti et al., 2014; Sixtus et al., 2018; Witt et al., 2010). This pattern mirrors the findings in language comprehension research, where both interference and facilitation effects have been reported. However, to date, a clear and encompassing theory for these patterns still seems to be missing (Ostarek & Huettig, 2019). Therefore, the role of experientially grounded meaning aspects during lexical selection remains unclear.

## Combining experientially grounded meaning aspects with distributional semantics

The so-called hybrid models are theories of semantic memory which integrate accounts of meaning based on experiential grounding with accounts based on distributional semantics. Theories of distributional semantics assume that the statistical regularities in natural languages are taken up by the cognitive system and are transferred into semantic representations which reflect the use of language (see below for more detail). According to the distributional hypothesis “you shall know a word by the company it keeps” (Firth, 1957), the meaning of a word can be deduced by the linguistic context in which it occurs. This idea has been implemented in different kinds of computational models quantifying meaning similarity between words by computing co-occurrence vectors (for an overview, see, e.g., Günther et al., 2019; Sahlgren, 2008; Wingfield & Connell, 2019). While implementations of distributional semantic models approximate human performance in many different tasks, they lack psychological plausibility as they cannot explain how concepts acquire meaning, which has also come to be known as the symbol grounding problem (Harnad, 1990; Searle, 1980). However, experiential accounts of meaning tend to disregard the importance of non-sensory and non-motoric sources of semantic knowledge.

Theories of distributional semantics and theories of embodiment or experiential grounding of semantics have often been treated as separate while a combination of these accounts in fact helps our understanding of semantic memory (Davis & Yee, 2021). Given that we learn concepts not only from direct sensory experience but also merely by being immersed in language given a sufficiently large directly grounded vocabulary (Louwerse, 2018), it becomes evident that the often-conceived gap between language-based distributional models of semantics and experientially grounded accounts of meaning is more dichotomous than necessary. Language use as reflected in large text corpora captures many aspects of our bodily and sensory experiences as we use language to communicate about them (Durda et al., 2009) and therefore, sensorimotor contingencies are not only a part of our direct experience but are also mirrored in distributional language use (Zwaan & Madden, 2005). Furthermore, we are able to learn about bodily and sensory experiences merely by being exposed to linguistic descriptions of these without firsthand experience but still yielding typical effects of experiential reactivation (Günther et al., 2018, 2020), pointing to the fact that oral and written language can in fact serve as just another source of experience. These observations lead to several calls for reconciling grounded and distributional accounts of meaning (Andrews et al., 2014; Davis & Yee, 2021).

In summary, language is used to communicate about the world and our experiences in the world and therefore it is not independent from it. Distributional semantics which rely on the statistical regularities in language use, therefore, often contain information about sensorimotor experiences (Louwerse, 2011). However, the correspondence between the direct sensorimotor experiences of the physical world and the experiential information extracted from language use alone is not 1:1. There are meaning aspects which can only be inferred from one of these sources (for a detailed discussion of the relation between sensorimotor grounded meaning and distributional semantics, see Günther et al., 2019) and at least part of our mental lexicon needs to be directly grounded (Vincent-Lamarre et al., 2015). This claim is backed up by increasing evidence that sensorimotor and distributional linguistic meaning aspects are interacting but distinct types of knowledge.

For example, Carota et al. (2021) found a widely distributed network of active brain regions during silent reading. Importantly, activity in brain regions relevant for semantic selection and combinatorial semantic processes correlated with a distributional model of the stimulus set while cortical regions associated with sensorimotor processing responded more strongly to the experience-based characteristics of the stimulus set.

While it is acknowledged that insights into the nature of semantic representations—which have mostly been gained by investigating language comprehension—should be incorporated into language production research (Vinson et al., 2014), neither theories based on distributional language usage nor experientially grounded theories—or a combination of both—played an important role in the investigation of lexical selection processes. Only recently, Banks and colleagues (2021) asked participants to produce category members for given semantic categories. They found that both the order and the frequency of produced words can be predicted by the measures of linguistic and sensorimotor similarity. These findings were also integrated into a computational model which performed most accurately with indirect spread of activation between categories and when sensorimotor and linguistic distributional aspects of meaning were accounted for. This is one of the first pieces of evidence suggesting that speakers make use of the experiential and linguistic contexts in which words occur and that they contribute separately when it comes to lexical selection.

However, an explicit integration of various aspects of meaning in language production models is still lacking (Abdel Rahman & Melinger, 2019; Vinson et al., 2014) and we know little about the role of distinct types of information from which word meaning can be learned (Louwerse, 2018; Vigliocco et al., 2009).

## The present study

This study was designed to test whether experientially grounded meaning aspects have an influence on which words we select when we prepare to speak and in how far they are influenced by distributional aspects of meaning. We combined the existing paradigms from the comprehension literature which show that physical visual stimulation has an influence on the processing of spatially connotated words (Berndt et al., 2018; Dudschig et al., 2013; Kaschak et al., 2005; Meteyard et al., 2008; Ostarek & Vigliocco, 2017) with the evidence for automatic reactivation of spatial meaning when processing up- and down-related words and sentences (Bergen et al., 2007; Dudschig et al., 2012; Estes et al., 2008; Lachmair, Dudschig, et al., 2016a; Lachmair et al., 2011; Ostarek et al., 2018; Öttl et al., 2017; Thornton et al., 2013; Vogt et al., 2019). We developed a paradigm which enables us to investigate whether activations of language-space associations—for which there is ample evidence in language comprehension—can be found in language production, too. To this end, we employed a free production task and manipulated both the visual presentation mode (upward vs downward movement of sentences) and the spatial content of the stimulus sentences (describing different locations in space). In contrast to previous studies investigating the duration of lexical selection processes (e.g., Hustá et al., 2021), we asked WHICH words are selected based on the contexts that pose little or no semantic constraints. Participants were asked to complete written sentence fragments (e.g., “You are strolling across the field and you see . . .”) by orally producing a noun of their choice. The fragments extended upwards or downwards from the centre of the screen, with each word being presented above or below the previous word.

As visual input has been shown to influence the processing of words with spatial connotations, we assumed that visual stimulation also influences lexical-semantic processing during language production. We expected lexical choices for completing the sentence fragments to be influenced by visuo-spatial manipulation; that is, the location of the produced nouns should be predicted by the upward or downward movement of the sentence fragments. In other words, participants should complete a sentence like “You are hiking through the forest and you see a. . .” with a noun like “bird”, referring to an entity that is typically found in the sky, after having read an ascending sentence, and with a noun like “fox” after having read a descending sentence.

In addition, we examined influences of the spatial location of the situation described by the sentences, investigating whether the typical location of the produced nouns can be predicted by the spatial connotations of the sentences. After sentences denoting situations which are perceived as occupying a higher physical space, such as “You are in the mountains . . .”, we expected nouns to refer to entities in the upper sphere and vice versa.

Moreover, we estimated the degree of semantic similarity between the produced noun and the noun in the sentence fragment using cosine values as a distributional measure of similarity (Günther et al., 2015). Semantic similarities are computed based on text corpora, and meaning relations of words that tend to occur in similar texts may capture different semantic relations as categorical, associative, or thematic links (Durda et al., 2009). Therefore, we used the distributional measure of similarity to obtain an estimate of semantic relatedness that captures the traditionally investigated semantic relations known to influence lexical selection during language production in semantic context paradigms. By relating our experiential spatial manipulations to a measure of semantic relatedness, we addressed the question of how experientially grounded and linguistic distributional semantic meaning aspects relate to each other in a production task with given sentence contexts.

## Experiment 1

### Methods

#### Participants

We recruited 35 native German speakers using the institutes’ participant pool Psychologischer Experimental Server Adlershof (PESA). The data of two participants were removed prior to analysis due to a high number of missing or invalid answers (less than 60% of remaining trials). The final sample consisted of 33 participants (24 females, 18–33 years of age, *M*_age_ = 25.82, *SD*_age_ = 4.56) who provided written informed consent prior to participation. The study was conducted according to the principles expressed in the Declaration of Helsinki and was approved by the local Ethics Committee. Participants received either course credit or monetary compensation.

#### Stimuli

In total, 90 German sentences, such as “*Du spazierst über das Feld und siehst eine . . .*” (English: “You are strolling across the field and you see a . . .”) or “*Du gehst zu der Haltestelle und siehst einen . . .*” (English: “You are walking towards the bus stop and you see a . . .”), were used as stimuli. All sentences had a similar structure and were incomplete. The first position of each sentence consisted of the personal pronoun “you”. At the second position, 30 verbs of motion (of which nine were stative verbs, e.g., “walk”, “stroll”, “run”, “sit” and “stand”) were used; thus, each verb appeared in three different sentences. The third position consisted of a local preposition, followed by a definite article at the fourth position. The fifth position constituted a noun containing the relevant information regarding the scene of the described event. Nouns were only used once and referred to an individual’s destination or places where a person can move around (e.g., “street”, “field”, “bus stop”, “forest”, “lake” and “train station”). The sentences continued with the conjunction “and” at the sixth position and a verb of perception at the seventh position (“see”, “spot” and “discover”), each repeated 30 times across all sentences. At the eighth position, there was an indefinite article. As accusative articles in German signify gender, we counterbalanced the distribution of neutral, female, and male articles over six experimental lists, assuring that each sentence was paired with each article equally often across participants and experimental conditions. After the indefinite article, the sentences ended with an ellipsis to prompt participants to complete the sentence. We ensured that a wide range of endings was possible for each sentence, that is, sentences were not constraining as, for example, in cloze paradigms (Block & Baldwin, 2010). We used 40 filler sentences with a similar structure as our experimental sentences. The ending for some fillers was intended to be more easily predictable to make the task easier for participants. Six additional sentences were used in practice trials.

##### Sentence spatial location

Before starting the main experiment, we conducted an online rating of the spatial locations of our sentences using the platform https://www.soscisurvey.de. Nine voluntary participants who did not take part in the main experiment (6 females, 22–67 years of age, *M*_age_ = 31.78, *SD*_age_ = 13.63) indicated on a 7-point Likert-type scale where the places denoted by the noun at the fifth sentence position are in space (see below for more information on spatial ratings). These values served as a measure of the spatial location of the scenes denoted by the sentences. These values were later added into our analysis to analyse the impact of the sentence location on the choice of a suitable sentence ending. A list of all sentences with their respective spatial location values is presented in the online Supplementary Material 2A.

#### Procedure

Before starting the experiment, we told our participants that we were investigating language processing of speakers with different native languages (Arabic, Chinese, and German) as a cover story. We deemed it common knowledge that Arabic and Chinese differ from German regarding reading direction and wanted to keep participants from wondering why stimuli were presented in an unusual reading direction to minimise the risk of participants guessing the aim of the task.

Participants were seated in a dimly lit room approximately 70 cm in front of a computer screen with a resolution of 1,280 × 1,024 pixels. Sentences were displayed consecutively in Rapid Serial Visual Presentation mode using Presentation^®^ software (Version 17, Neurobehavioral Systems, Inc., Berkeley, CA, www.neurobs.com). The experiment started with a practice block consisting of six sentences. During the experiment, participants were able to take small breaks after blocks of 15 sentences.

**Figure 1.**
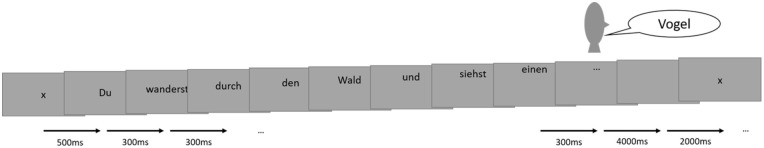
Trial sequence with an example of an upward moving sentence (English: “You are walking through the forest and you see . . .”) and a participant producing the noun “bird”. Note that screen position was fixed in the experiment and only moves upwards in the figure for illustrative purposes.

Each sentence was presented once during the experiment and participants saw each sentence either in ascending or descending presentation direction. Filler sentences were presented in the same way. Within each list, test sentences and fillers were presented in a random order. We presented each word for 300 ms in black on a grey background in Arial 24 pt font. Each trial started with a fixation cross appearing in the centre of the screen for 500 ms. Then the first word appeared in the centre of the screen. The following word replaced the previous word and appeared either 35 pixels higher or lower than its predecessor. Thus, the position of the three dots was 315 pixels above or below the screen centre and 197 pixels apart from the edge of the screen. The dots remained on screen for 4,000 ms; afterwards, there was a blank screen for 2,000 ms before the next trial started. Participants were instructed to read the sentences silently and to complete the sentences with a suitable noun as spontaneously and quickly as possible as soon as the ellipsis appeared. Participants were asked to orally produce only one word in each trial and to avoid repeating the same noun several times throughout the experiment. We recorded answers given in the time frame of 6000 ms after the ellipsis appeared (see Figure 1 for illustration of a trial sequence). The experimenter monitored the experiment from another room and immediately noted the answers.

##### Rating of spatial attributes of produced nouns

In a second step, after running the sentence completion study, spatial attributes for the produced nouns were obtained to assess whether the choice of the produced nouns had been influenced by the experimental manipulation. To this end, the produced nouns entered a rating study. Nouns uttered by several participants were included only once (e.g., several people used the word “bird”, albeit some used it in different contexts throughout the experiment). Nouns which presumably have the same referent—but where participants used different lexemes to convey a comparable meaning—entered the rating in all the forms which had been produced during the experiment (e.g., “*Schiffsanleger*” vs “*Bootsanleger*,” English roughly “jetty” vs “pier”). In case of ambiguous nouns, a short description of their lexical meaning was added. For example, as could be inferred from the context, the word “*Sirene*” had not been intended to refer to the English “siren”, but to the mythological figure of a mermaid. Therefore, raters saw this noun as “*Sirene (Fabelwesen)*”, English: “siren (mythological figure)”. The complete set of produced words was reduced to a set of 1,056 rated words implemented similarly to Díez-Álamo et al. (2018) and Scott et al. (2019). To reduce the time for the rating for each rater, each rater only saw a subset of the total word set. To this end, the produced nouns were randomised and then distributed on 21 questionnaires, with the first questionnaire containing words 1–352, the second questionnaire containing words 51–402, the third questionnaire containing words 101–452, and so on, thereby ensuring that the questionnaires were representative of the whole set of produced nouns. In addition, 20 control words were added to each questionnaire. These control words had not been produced in the sentence completion study but were taken from an unpublished set of spatial ratings for German nouns, spanning the entire range of locations where entities can be encountered, from very high up (“*Sternschnuppe*”, English: “falling star”) to very low (“*U-Boot*”, English: “submarine”). The questionnaires were administered using the online platform https://www.soscisurvey.de. In each trial, a target word was selected randomly and displayed with a vertical 7-point rating scale ranging from *up* to *down* (with “centrally” at the midpoint) below it. In addition, participants could skip the rating of a word in case the spatial property could not be judged. By clicking one of the points on the scale, participants had to judge where the object referred to by the noun can typically be found. The approximate time to complete the rating was 20 min. In total, 37 voluntary participants who did not take part in the production experiment (22 females, 23–59 years of age, *M*_age_ = 35.76, *SD*_age_ = 9.61) were randomly assigned to one of the questionnaires. The procedure of assigning different questionnaires to participants ensured that each target word received ratings from at least nine subjects. Assuming that ratings for control words whose spatial location was based on previous rating data are an indicator of subject’s compliance, intra-class correlation between the previous rating data and each rater was computed using the function ICC from the R-package psych (Revelle, 2018), as suggested by Hallgren (2012) and Trevethan (2017). The agreement with the existing mean rating values for the control words was *ICC*(3,1) ⩾ .72 for all raters and the intra-class correlation between all subjects was excellent following the criteria of Fleiss (1986), ICC(3,1) = .83. Therefore, none of the ratings were excluded, and ratings for all but the control nouns were averaged across raters, yielding one rating value for each distinct noun. This served as an indicator of the spatial location of the entity denoted by that noun. Ratings were merged with the data from the sentence completion study so that for each trial, a mean spatial rating serving as an indicator of the spatial location of the produced noun was obtained.

### Data analysis

Data were analysed using the free statistics software R Version 3.6.1 (R Core Team, 2017). The data set consisted of 2,970 data points (33 participants completing 90 sentences). Missing trials in which participants did not produce a noun were excluded (12.2% of all trials). Afterwards, erroneous trials (incomplete, unintelligible, and nonsensical answers and utterances which consisted of more than one word or in which participants simply repeated the noun of the sentence which they had read) were excluded from further analysis (2.9% of all trials). In addition, eight trials had to be excluded for missing spatial rating values due to experimenter error. Trials in which participants produced nouns whose gender did not match the gender required by the article were not excluded from analysis, as some participants seemed to have ignored the gender of the article. There were many instances of masculine nouns being produced after neutral articles, which is incorrect from a grammatical point of view. However, the masculine accusative article “*einen*” is typically shortened to “*ein*” in colloquial speech, equaling the neutral article. Thus, it cannot be safely concluded that participants ignored the gender of the article in those cases, as they might have silently pronounced the written sentences before giving an answer aloud. The phonological similarity of “*einen*” and “*ein*” in spoken German might have led them to produce nouns of both neutral and male gender, respectively. In total, a set of 2,515 utterances remained for statistical analysis.

To assess the influence of the experimental manipulation on the spatial properties of the produced nouns, a linear mixed model was computed with the packages lme4 (Bates et al., 2015) and lmerTest (Kuznetsova et al., 2017). We started with a maximal model containing interactions between the fixed predictors presentation direction and centred spatial location values for the nouns of the sentence fragments, and by-subject and by-item random intercepts and slopes. Sliding difference contrasts were applied for the predictor presentation direction. Random effects were simplified in case of singular fit or convergence problems, resulting in the final model containing by-subject and by-item random intercepts only. Using model comparison, this model was compared with one containing additive fixed effects for presentation direction and centred spatial location values. We report *beta*-estimates together with a 95% confidence interval estimated with the Wald method, and *t*- and *p*-values.

### Results

**Figure 2.**
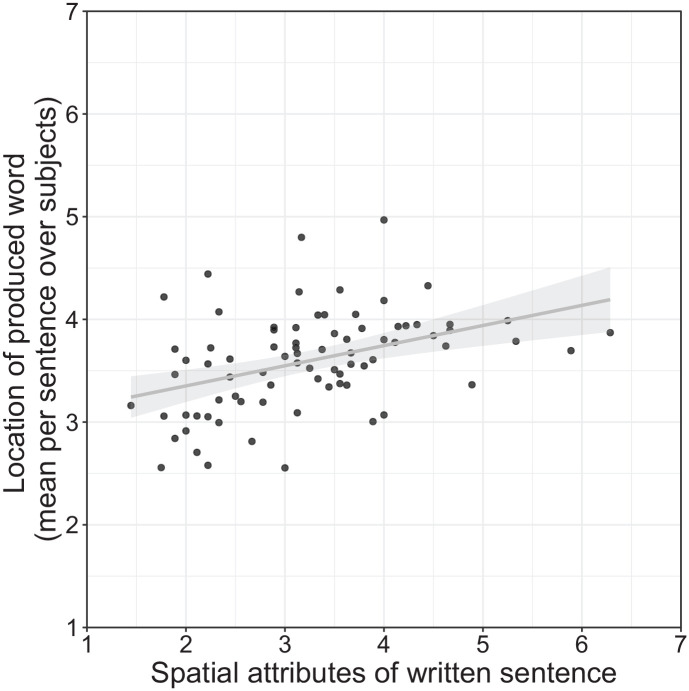
The spatial location of nouns in the sentence fragments predicts the location of the noun referents chosen as suitable sentence endings. The line depicts the effect as estimated in the linear models, the dots represent mean spatial values of the produced words for each sentence fragment, respectively. Spatial locations of the entities referred to with the produced nouns were rated on a scale ranging from 1 (down) to 7 (up) after the experiment. Spatial locations of nouns in the sentence were rated on a scale ranging from 1 (down) to 7 (up) before the experiment. For illustrative purposes, sentence noun spatial locations are not centred.

Numerically, there was almost no difference in mean spatial ratings between nouns produced after ascending versus descending sentences (*M*_up_ = 3.577, *M*_down_ = 3.582). This finding was corroborated using a linear mixed model containing additive effects for presentation direction and spatial location values for stimuli, which explained the data better than a model containing interactions, χ^2^(1) = 0.971. There was no main effect for presentation direction (β = −.01 [−.10, .07], *t* = −0.26, *p* = .80), but there was a significant main effect of sentence spatial location (β = .20 [.11, .28], *t* = 4.69, *p* < .001), indicating that the spatial locations of the situations presented by the sentence fragments influenced the spatial attributes of the produced nouns, see Figure 2.^1^

To gain further insights into the relation between this effect and traditionally investigated semantic measures known to affect conceptual-semantic processing during language production, we included a distributional measure of semantic similarity between the nouns in the presented sentences and the produced nouns as a covariate in the analysis. We used the semantic space dewak100k_cbow (Günther et al., 2015) built from the deWaC-corpus using the cbow algorithm as implemented in the word2vec model (Mikolov et al., 2013). The deWaC-corpus is a 1.7 billion word corpus constructed from the Web limiting the crawl to the .de domain and using medium-frequency words from the Süddeutsche Zeitung corpus and basic German vocabulary lists as seeds (Baroni et al., 2009). Cosine values were computed for each pair of fifth sentence position and produced nouns using the package LSAfun (Günther et al., 2015). These cosine values serve as an indicator of semantic similarity, with higher values indicating that the two respective words more often occur together in similar contexts than others and have a highly similar meaning.

**Figure 3.**
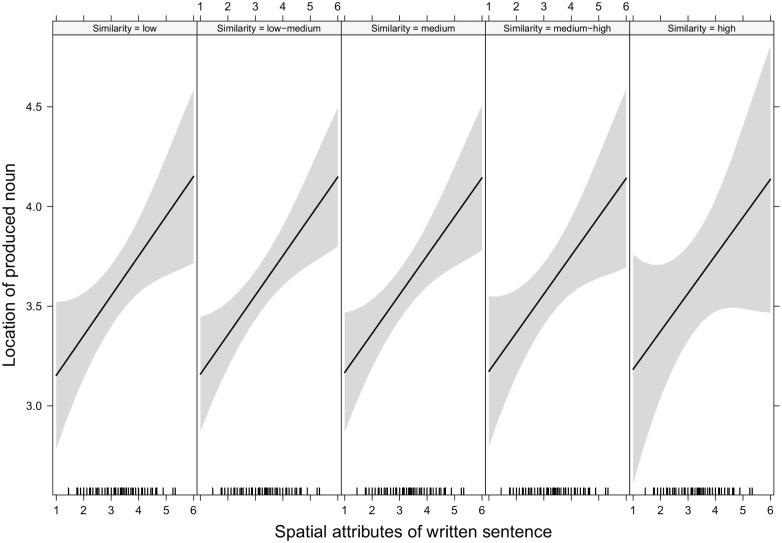
Effect plot showing that increasing degrees of semantic similarity between the noun in the presented sentence and the produced noun did not influence the spatial attributes of the presented sentences. Higher values for location of the produced noun and for spatial attributes of the written sentences indicate a higher localisation in space. Small ticks above the x-axis mark the distribution of the set of sentences regarding their spatial properties. For illustrative purposes, sentence spatial locations are not centred. The continuous predictor of similarity was split into five points of equal distance. Low and high similarity refer to the lowest versus highest cosine values obtained in this study; they are used as descriptive labels while no pre-defined level of degrees of semantic similarity regarding cosine values exists.

Because not all words were included in the used corpus, cosine values could not be computed for all cases. Furthermore, trials with cosine values less than zero were not used for subsequent analyses as these cosine values cannot be interpreted in a meaningful way (Günther et al., 2015). Thus, the reduced data set with similarity measures consisted of 1,904 out of 2,515 total nouns which had been used for the first linear mixed model analysis. Centred cosine values were entered into the linear model as an additional predictor with main effects for direction and an interaction between cosine values and centred sentence spatial location values, and random intercepts for items and subjects. There was no interaction between semantic similarity as indexed by cosine values and sentence spatial location values (β = .08 [−.35, .50], *t* = 0.36, *p* = .72). Thus, the effect of sentence spatial location on spatial properties of the produced nouns cannot be explained by similarity, see Figure 3.

Again, there was no effect of direction but a significant main effect of sentence spatial location (β = .20 [.10, .29], *t* = 4.07, *p<* .001). For similar results obtained with the semantic space de_wiki, see Supplementary Material 1 Table S1.

### Discussion

There was no influence of the manipulation of presentation direction on the spatial properties of the produced nouns. Therefore, our hypothesis that visual spatial manipulation in the form of a physical spatial cue affects lexical selection was not confirmed. However, there was an influence of the experientially driven meaning dimension “location in space” on the choice of nouns. When considering the typical spatial location of the situations described by the sentence fragments, the spatial properties of the produced nouns could be predicted. Thus, the more a sentence referred to a situation in the upper or lower domain of the world, the higher up or lower down the referents of the produced nouns were located. For example, after the sentence “You lean at the window and you see a . . .” which had been rated as being found in the upper sphere, the nouns people produced tended to be more upwards related like “bird” or “rainbow” as when people completed sentences like “You jump over the tree trunk and you see a. . .” which had been rated as being in the lower sphere and where people were more likely to produce words as “rainworm” or “hole” which are also more downwards related in comparison with upwards-related words like “bird’s nest”. This might demonstrate that experiential traces of space are reactivated during language processing and influence subsequent lexical selection. We will discuss this interpretation in the “General Discussion” section.

However, many participants reported that the task was difficult for them, reflecting the high number of lost and invalid trials (15.1%, see “Methods” section). We had aimed to prevent participants from preparing a possible answer prior to reaching the end of the sentence by also presenting an indefinite article. As German articles determine gender in the accusative case, participants had to wait until they read the article before a lexical choice could be made. Thereby, we wanted to maximise the impact of the visual manipulation and prevent participants from preparing their answer in advance. However, this manipulation made it difficult to come up with suitable nouns, as time for completing a sentence was limited and led to omissions, neglecting the case of the article, or—as in about 20% of all trials—naming a person. This was a wide-spread strategy to fulfil the gender requirements of the article. For instance, participants could say “*Polizist*” in case of the male article “*einen*” (English: “policeman”) and “*Polizistin*” in case of the female article “*eine*” (English: “ policewoman”). However, naming a person is not informative about the spatial attributes of a noun, as persons are usually found in the central plane and occupy the same space as a person experiencing the situation described by the sentence. The large number of trials in which a noun referring to a person was produced may have reduced the impact of the movement manipulation. In Experiment 2, we therefore presented sentence fragments with no articles and asked participants to not name a person.

## Experiment 2

Experiment 2 was a preregistered study using the Open Science Framework (https://osf.io/se6a3/?view_only=f666716d3b8f47228017b9dadc6e2950) designed to replicate the findings of Experiment 1. To reduce task complexity and trial loss, words were presented for slightly longer and sentences did not end with an article. Thus, participants were asked to produce a determiner noun phrase and were not restricted in their selection of suitable nouns regarding gender. Furthermore, we introduced a baseline condition in which sentences were presented in the centre of the screen. We also improved the stimulus set by balancing the sentence spatial location values of the sentence fragments across the different presentation directions. In addition, the number of participants was increased to enhance the chances of detecting even small effects of the movement manipulation on lexical selection.

### Methods

Only those aspects differing from the first experiment will be described below.

#### Participants

We recruited 78 native German speakers.^2^ Data of two participants were removed prior to analysis as their German language proficiency was limited despite reporting being native speakers. Furthermore, the data of four other participants were excluded due to not following the instructions (*N* = 2) or a high number of missing or invalid answers (*N* = 2). The final sample consisted of 72 participants (49 females, 18–35 years of age, *M*_age_ = 25.60, *SD*_age_ = 4.93). Participants provided written informed consent prior to participation. The study was conducted according to the principles expressed in the Declaration of Helsinki and was approved by the local Ethics Committee. Participants received either course credit or monetary compensation.

#### Stimuli

In total, 60 German sentences with a similar structure as in the first experiment were used as stimuli, for example, “*Du läufst zum Feld und siehst . . .*” (English: “You are walking towards the field and you see . . .”). Compared with the first experiment, only four verbs of motion (“stand”, “walk”, “go”, and “enter”) and the verb “be” were used at the second position, appearing equally often across the full set of sentences. The third position consisted of a definitive article or a local preposition contracted with a definite determiner (e.g., “*am*”—“at the”, “*zur*”—“towards the”). At the fourth position, a noun conveying the relevant information about the location at which the scene happened was used. The sentences finished with the conjunction “and” at the fifth position and a verb of perception at the sixth position. The sentence display terminated with an ellipsis “. . .”, serving as a prompt for the participants to complete the sentence with a suitable noun. Furthermore, we constructed 24 filler trials with a similar structure and the same number of words as the sentences. Six additional sentences were used in practice trials.

##### Sentence spatial location

Before starting the main experiment, we conducted an online rating of the spatial location of our sentences using the platform https://www.soscisurvey.de. Overall, 15 voluntary participants who did not take part in the main experiment (8 females, 27–71 years of age, *M*_age_ = 34.00, *SD*_age_ = 10.76) indicated on a 9-point Likert-type scale where the places denoted by the noun at the fourth sentence position are in space.^3^ Apart from adding them as predictors into our analysis, the sentence spatial location values were used to construct experimental lists. All sentences with their respective sentence spatial location values are presented in the online Supplementary Material 2B.

#### Procedure

Mean rating values were computed for each of the sentence nouns ranging from 2.4 (“*Kanal*”*/*“canal”) to 7.7 (“*Aussichtspunkt*”/“vantage point”). Afterwards, three sentences with nouns of a similar mean rating value were combined into a triplet with the goal of minimising the difference in mean rating values between nouns in the same triplet. The resulting difference was 0.31 or less, with a mean difference of 0.08 between the nouns of an adjacent sentence pair. Each participant read each sentence fragment from each triplet. Each participant saw each sentence of a triplet only once in one of the three presentation directions. Presentation direction and triplets were counterbalanced over nine lists so that every sentence was presented equally often in the same direction across participants. This resulted in every participant reading sentence fragments with similar spatial location values in each condition. Thereby, we controlled potential impacts of spatial locations on our stimuli with regard to the manipulation of presentation direction.

Each trial started with a fixation cross presented for 500 ms, after which each word was presented for 400 ms. Each sentence ended with an ellipsis, serving as an indicator that participants should complete the sentence. After 4s, a circle was presented in the centre of the screen. Then participants could start the next trial in a self-paced manner by pressing the space bar on the keyboard. Responses were recorded within a window of 6 s after the appearance of the ellipsis. The first word of a sentence always appeared in the centre of the screen. The following word appeared either at the same position, 47 pixels above or below that original position, or replaced the previous word at the centre. The position of the ellipsis was 329 pixels above or below screen centre and 183 pixels apart from the edge of the screen. Participants were instructed to read each sentence fragment and to spontaneously produce a noun to end the sentence as quickly as possible and, if necessary, with appropriate determiner. We asked them to not complete the sentences by repeating parts of the sentence or by producing nouns describing a person (e.g., “a woman”, “a bus driver”, and “a neighbour”). The experimenter monitored the experiment in the same room behind a folding screen, taking notes of participants’ answers.

##### Rating of spatial attributes of produced nouns

To minimise the total number of words entering the rating, nouns with the same—or very similar—referents entered the rating only in one form, assuming that the spatial properties of the referents of these almost synonymous words would be the same. In addition, nouns produced several times by different participants entered the rating only once.^4^ Thus, the total number of produced nouns was reduced to 915 words divided into 10 questionnaires, each containing 457 or 458 words and 20 additional control words. Data from one rater were excluded prior to computing mean spatial ratings, as the intra-class correlation coefficient with the control words—which was only in the fair range, *ICC*(3,1) = .58—indicated that this participant did not follow the instructions. Therefore, data from 30 participants (19 females, 18–76 years of age, *M*_age_ = 33.57, *SD*_age_ = 13.82) were used to compute mean spatial rating values, with each target word having been rated by at least 13 subjects. Mean spatial ratings were merged with the data from the sentence completion study to obtain a numeric indicator of the spatial location of each produced word.

### Data analysis

The data set consisted of 4,320 data points (72 participants completing 60 sentences). Trials in which participants did not produce a noun (2.4% of all trials), erroneous trials (1.4% of all trials) and trials in which participants produced a noun describing a person (4.2% of all trials), were excluded. In addition, 13 trials for which no spatial ratings were obtained due to experimenter error were excluded from further analysis. Based on our preregistered criteria, all trials including the sentence “You are at the harbor and you see. . .” were excluded from analysis because more than 50% of the participants chose the same noun to complete the sentence. After pre-processing, the data set consisted of 3,893 nouns.

Like Experiment 1 and based on the preregistered analysis plan, we analysed the data with a maximal model containing interactions between the fixed predictors presentation direction and centred spatial location values for the nouns of the sentence fragments and by-subject and by-item random intercepts and slopes. Sliding difference contrasts were applied for the predictor presentation direction (three levels: descending, central, and ascending). Random effects were simplified in case of singular fit or convergence problems which resulted in the final model containing by-subject and by-item random intercepts only. Using model comparison, this model was compared with one containing additive fixed effects for presentation direction and centred spatial location values.

### Results

**Figure 4.**
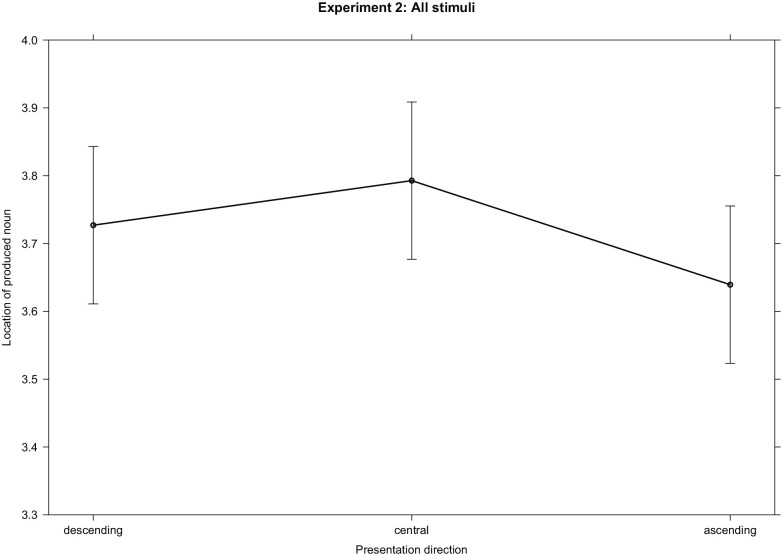
Estimated means and 95% confidence band for spatial locations of the produced nouns depending on the presentation direction of sentence fragments in Experiment 2.

**Figure 5.**
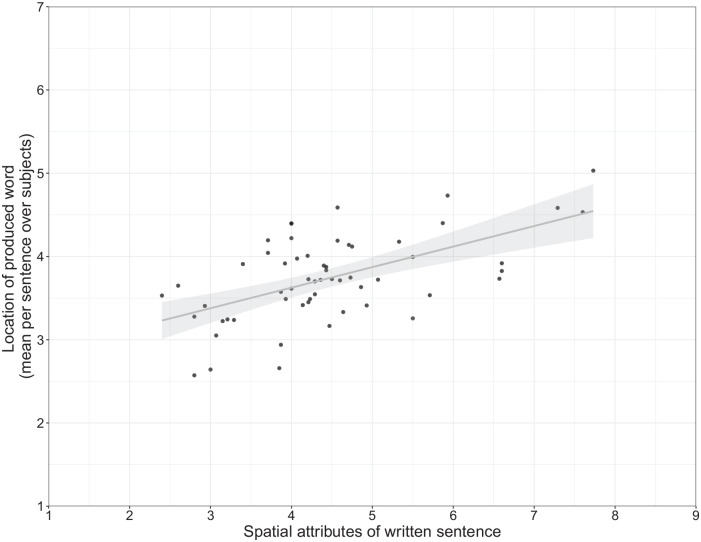
The spatial location of nouns in the sentence fragments predicts the location of the noun referents chosen as suitable sentence endings. The line depicts the effect as estimated in the linear models, the dots represent mean spatial values of the produced words for each sentence fragment, respectively. Spatial locations of the entities referred to with the produced nouns were rated on a scale ranging from 1 (down) to 7 (up) after the experiment. Spatial locations of nouns in the sentence were rated on a scale ranging from 1 (down) to 9 (up) before the experiment. For illustrative purposes, sentence noun spatial locations are not centred.

As in Experiment 1, a model containing additive effects for presentation direction and spatial location values for stimuli explained the data best, χ^2^(2) = 0.876. Contrary to our hypothesis, nouns produced after ascending sentences were located lower in space (*M_up_* = 3.64) than nouns produced after sentences with unchanging position (*M*_central_ = 3.79), resulting in a significant main effect for the contrast of ascending versus central presentation direction in the linear mixed model (β = −.15 [.24, −.07], *t* = −3.51, *p* < .001). There was no significant main effect for the contrast of descending versus central presentation direction (β = .07 [−.02, .15], *t* = 1.50, *p* = .133), see Figure 4. Furthermore, and converging with results from Experiment 1, there was a significant effect for sentence spatial location values (β = .28 [.19, .37], *t* = 6.32, *p* < .001), indicating that the locations of the sentence fragments influenced the spatial attributes of the produced nouns, see Figure 5.

For comparison with Experiment 1, an additional linear model was fitted post hoc to allow for a direct comparison of ascending versus descending presentation direction. Nouns produced after ascending sentences were located lower in space (*M_up_* = 3.64) than nouns produced after descending sentences (*M*_down_ = 3.73), as demonstrated by a significant main effect for the contrast of descending versus ascending presentation direction in the linear mixed model (β = −.09 [.17, .00], *t* = −2.01, *p* = .045).

**Figure 6.**
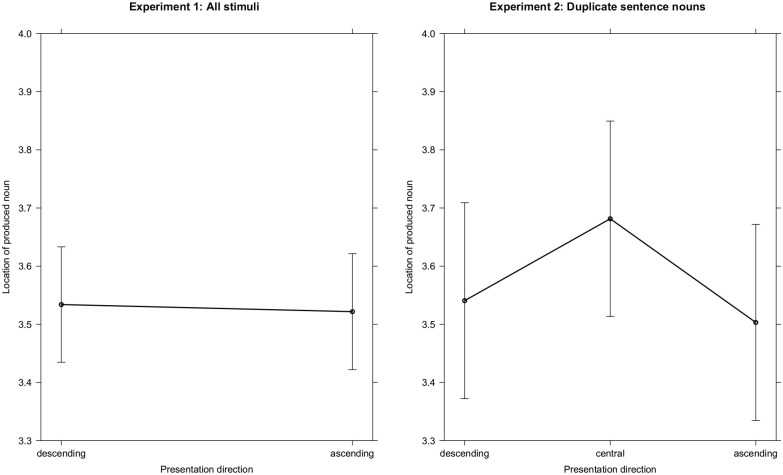
Estimated means of spatial properties of produced nouns depending on the presentation direction of the presented sentences and 95% confidence intervals. There is no statistical difference between ascending and descending presentation direction in Experiment 1 (left panel) and when investigating the 27 overlapping stimuli from both experiments (right panel).

The differential outcomes of Experiments 1 and 2 were further investigated by comparing results from the subset of sentences with overlapping noun use between experiments (27 out of 60 sentences). For the subset of sentences from Experiment 2, linear mixed models were specified as above without random intercepts for subjects due to singular fit. This again resulted in a significant difference between ascending and central presentation direction (β = −.18 [−.32, −.04], *t* = −2.47, *p* = .014), and a marginally significant difference between central and descending presentation direction (β = .14 [.00, .28], *t* = 1.95, *p* = .051) and a significant effect for sentence spatial location values (β = .31 [.16, .46], *t* = 4.16, *p* < .001). In comparing ascending and descending presentation direction directly, no significant difference between ascending and descending presentation direction was obtained (β = −.03 [−.18, .10], *t* = −0.52, *p=* .605), see Figure 6. Therefore, the difference between ascending and descending presentation direction when analysing the complete data set of Experiment 2 seems to hinge on items exclusively used in Experiment 2.

**Figure 7.**
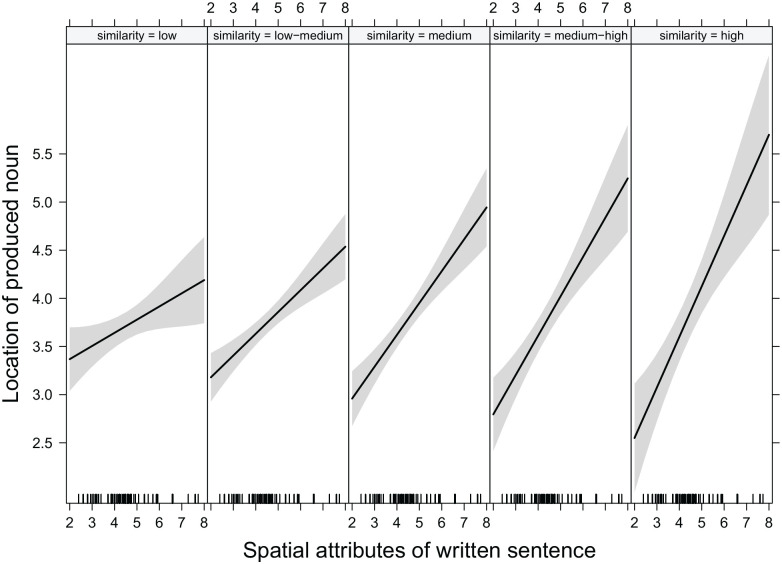
Effect plot showing more pronounced influences of the spatial attributes of the presented sentences on the spatial characteristics of the produced nouns for increasing degrees of semantic similarity between the noun in the presented sentence and the produced noun. Higher values for location of the produced noun and for spatial attributes of the written sentences indicate a higher localisation in space. Small ticks above the x-axis mark the spatial property distribution of the set of sentences. For illustrative purposes, sentence spatial locations are not centred as they were in the analysis. The continuous predictor of similarity was split into five points of equal distance. Low and high similarity refer to the lowest versus highest cosine values obtained in this study. They are used as descriptive labels, while no pre-defined level of degrees of semantic similarity with regard to cosine values exists.

As in Experiment 1, semantic similarity measures were included in the model to test if the pattern in our data was influenced by the semantic similarity between the content in the displayed sentence and the produced noun. Therefore, cosine values were computed for each pair of nouns (one at the fourth sentence position and the one being produced) in the respective sentence using the semantic space dewak100k_cbow. Cosine values could be computed for 3,570 trials out of 3,893 which had entered statistical analysis. Centred cosine values were added to the linear model as an additional predictor with a main effect for direction and an interaction between cosine values and sentence spatial location values, and random intercepts for items and subjects. Again, there was a significant main effect of sentence spatial location (β = .27 [.18, .36], *t* = 5.79, *p* < .001) and a significant difference between central and ascending presentation direction (β = −.10 [.18, −.01], *t* = −2.15, *p* = .032). In addition, there was a significant interaction between semantic similarity as indexed by cosine values and sentence spatial location values (β = .54 [.26, .81], *t* = 3.86, *p* < .001), indicating that the effect of sentence spatial characteristics was influenced by the degree of semantic similarity between the sentence noun and the produced noun, see Figure 7. For similar results obtained using the corpus de_wiki, see supplementary Material 1 Table S2.

We further investigated this interaction by splitting the range of obtained cosine similarities in between the highest and lowest similarity values in equally distant ranges. Then, we explored whether the effect of spatial characteristics of sentences on the produced nouns is contingent on a certain level of semantic similarity or if it exists across the entire range of semantic similarities.^5^ Taking all trials from each level of similarity (low, low–medium, medium–high, and high) into account, separate linear mixed models with the fixed predictors presentation direction, centred spatial location, and random intercepts for items and subjects were computed. In case of singular fit, random effect structures were simplified. As shown in Table 1, the effect of spatial characteristics of sentence locations on spatial locations of produced nouns is significant for each level of semantic similarity and gets more pronounced with higher degrees of semantic similarity between sentence nouns and produced nouns.

**Table 1.** Linear mixed model output statistics for the influence of presentation direction and sentence spatial location on the spatial properties of the produced nouns for different degrees of semantic similarity between the sentence noun and the produced noun based on centred cosine values.

Similarity range Examples	Low 0 < cos ⩽ 0.216 tree—lady bug, picture house—stars, swing lake—animal, towel *n* = 856				Low–medium 0.216 < cos ⩽ 0.366 tree—bicycle, swing house—fence, chair lake—inflatable mattress, fishing rod *n* = 1,454 no random intercept for subjects				Medium–high 0.366 < cos ⩽ 0.516 tree—bird, squirrel house—garage, window lake—pier, water lily *n* = 965 no random intercepts for subjects				High 0.516 < cos tree—branch, leaves house—hut, garden lake—boat, shore *n* = 295 no random intercepts for subjects			
Variable	*b*	95% CI	*t*	*p*	*b*	95% CI	*t*	*p*	*b*	95% CI	*t*	*p*	*b*	95% CI	*t*	*p*
Intercept	3.66	[3.51, 3.80]	49.27	<.001	3.72	[3.59, 3.85]	55.48	<.001	3.69	[3.50, 3.88]	38.04	<.001	3.70	[3.41, 3.98]	25.56	<.001
Direction (cent-down)	−.14	[−.33, .04]	−1.51	.131	.04	[−.10, .18]	0.50	.618	−.09	[−.24, .05]	−1.24	.214	.04	[−.17, .25]	0.35	.726
Direction (up-cent)	.00	[−.19, .19]	0.00	.996	−.12	[−.26, .02]	−1.64	.102	−.12	[−.26, .03]	−1.58	.114	−.13	[−.34, .09]	−1.15	.251
Sentence location	.20	[.08, .32]	3.31	.002	.22	[.11, .33]	4.03	<.001	.25	[.09, .41]	3.14	.003	.49	[.23, .75]	3.67	<.001

CI: confidence interval.

To illustrate the range of cosine values, the non-centred equivalents of the cosine values on which analyses were based are given together with example pairs from the data set consisting of a noun which had been part of the presented sentence and two exemplars of nouns produced after these sentences. Analyses were based on all trials which fell in a certain range. The upper boundary of the low similarity range corresponds to the point value in the second column of Figure 7, the lower boundary of the low–medium range corresponds to the point value in the second column of Figure 7 and the upper boundary of the low–medium range corresponds to the point value in the third column of Figure 7, and so on.

Furthermore, we additionally explored in how far the observed effects of sentence spatial location on lexical choices hinged on predictability. To this end, we computed cloze values for the stimuli which ranged from 0.07 to 0.43 per sentence proving that none of the sentence endings was highly predictable. The absolute number of produced words per stimulus sentence ranged from 17 to 42 different words, see Supplementary Material 1 Table S3 for information on the predictability of words for each sentence from our stimulus material.

We then ran an additional post hoc analysis with only those sentences included where less than 36 different nouns had been produced as an answer (in total, 72 different nouns could have been produced potentially) which are the most highly predictable stimuli in the stimulus set. This reduced dataset with 40 out of 59 stimulus sentences yielded very similar results to the main analysis with a significant interaction between spatial location of sentences and semantic similarity, β_high-pred_ = .57, *t* = 3.28, *p* = .001.

On the contrary, when looking at those cases where participants had produced the most diverse answers, that is, 36 or more different nouns per sentence (19 out of 59 stimulus sentences), there was no interaction between spatial locations and semantic similarity, β_low-pred_ = .31, *t* = 1.43, *p* = .153, while the main effect for spatial locations of stimulus sentences on spatial locations of produced nouns was significantly evident in both subsets, β_high-pred_ = .24, *t* = 3.94, *p* < .001 and β_low-pred_ = .30, *t* = 6.05, *p* < .001. Thus, the effect of spatial locations of the stimulus material on spatial locations of the produced nouns persists when predictability is minimised.

### Discussion

We replicated the main effect of sentence spatial locations, finding that the referents of produced nouns were higher up/lower in the world when sentences described situations higher up/lower in the world, as indicated by prior ratings of the nouns in these written sentences. For example, when choosing suitable sentence endings for the sentence “You are at the vantage point and you see . . .”, participants chose words, such as “sky”, “mountains”, “skyscraper”, or “Ferris wheel” while they completed the sentence “You are at the canal and you see. . .” with words, such as “ant”, “stones”, or “litter”. By showing that spatial meaning traces influence the choice of words in an open language production task, we were able to demonstrate that experientially driven meaning aspects in the spatial domain have an impact on lexical selection during language production. Furthermore, there was an interaction between semantic similarity and sentence spatial location, indicating that the effect of sentence spatial location on the spatial properties of the produced noun was higher when the sentence noun and the produced noun were semantically related. In contrast to Experiment 1, there was a significant effect of the spatial manipulation on the spatial properties of the produced nouns. The different results in Experiments 1 and 2 concerning the influence of semantic similarity and the impact of presentation direction on the spatial properties of the produced nouns are discussed in the next section.

## General discussion

In two experiments, we investigated the role of experientially grounded meaning in language production. We manipulated the meaning dimension of “location in space” in two complementary context conditions in one of the two ways: (1) physically and isolated from the meaning of verbal contexts as a simulated ascending or descending movement or (2) embedded within verbal contexts. Specifically, participants read sentence fragments like “You hike through the forest and you see. . .” and completed them with a suitable noun of their choice. Starting from the centre of the screen, the words were presented in a simulated upward or downward movement, that is, a physical vertical visual manipulation. In addition, spatial cues were conveyed via the meaning of the sentences, that is, verbally referring to situations in different spatial locations, such as “You walk to the field and you see. . .” or “You are on the balcony and you see. . .”. We tested whether the physically or verbally transmitted spatial experiential manipulations affect our lexical choices.

### Experiential traces embedded in meaningful contexts, but not physical cues, lead to experientially grounded lexical selection

Contrary to the hypothesis that visual motion affects lexical selection, the physical simulation of visual motion did not influence which words participants chose in Experiment 1. The result was replicated in Experiment 2 when considering the set of sentence nouns which had already been used in Experiment 1. There were no differences in spatial characteristics for nouns produced after descending versus ascending sentences. The unexpected effect of spatial characteristics of produced nouns being higher after centrally presented sentences than after descending and ascending ones in Experiment 2 seems to be an artefact of this additional condition. The central condition differed from ascending and descending movement manipulations because sentences were presented statically without movement simulations involved. Furthermore, the difference between ascending and descending presentation direction for the whole set of stimuli in Experiment 2 seems to hinge on some of the newly introduced sentence nouns, as it was not existent for the set of items in Experiment 1. In addition, these effects were small compared with the effects of sentence spatial locations on produced nouns (to be discussed in detail below). Therefore, the unexpected effects for Experiment 2 are not reliably observed and may have been caused by the additional central sentence presentation and variations in stimulus material. Potentially, in future studies, a sentence display where the control condition is displayed with a slight shift movement to the right—and not statically—might help to clarify this issue. With regard to stimulus material, Experiments 1 and 2 differed as follows: stimulus sentences were presented with article (Experiment 1) and without article (Experiment 2), and the stimulus set in Experiment 2 was more generic with verbs not describing manner of motion as some verbs in Experiment 1 did (in Experiment 2, five different verbs had been used: “stand”, “walk”, “enter”, “go”, and “be”, whereas in Experiment 1, 30 different verbs had been used, among which verbs such as “balance”, “paddle”). Furthermore, the stimulus set was reduced to 60 stimulus sentences in Experiment 2 (Experiment 1: 90 stimulus sentences). We do not have an assumption why these differences may have led to the unexpected effect. The comparison of effects between Experiments 1 and 2 with identical situations described in the stimulus sentences (see Figure 6) suggests that not only the central presentation but also some differences in the stimulus material between Experiments 1 and 2 may have contributed to different outcomes. However, this may not generalise and should rather be interpreted as no support for an effect of visual spatial manipulations on lexical selection. New data from an experiment in which we investigated whether body posture changes influence lexical choices and where we used most of the stimuli from Experiment 2 further support this interpretation. In this study, there was a significant difference between nouns produced after upward head movements compared with downward head movements, in line with the hypotheses (Vogt et al., 2022).

The absence of an effect of physical cues speaks against a high susceptibility for experientially grounded aspects on lexical access during language production. This stands in contrast to empirical evidence for experientially grounded language comprehension where influences of visual cues on processing of sentences, nouns, and verbs have been reported in different paradigms (Dudschig et al., 2013; Kaschak et al., 2005; Meteyard et al., 2008). One possible explanation for the lack of comparable effects in language production is that the physical manipulation does not transport sufficient meaning to affect the lexical-semantic construction of verbal messages. Analogously, it has been shown that physical spatial cues alone are not sufficient to facilitate an anagram-solving task, whereas the combination of spatial and situational cues is (Berndt et al., 2018). Presumably, a higher task relevance of the physical manipulation leading to more effortful linguistic processing (Louwerse et al., 2015) and a stronger bodily involvement (i.e., by changing the body position as in the study by Lachmair et al., 2016b) may yield an effect of spatial manipulations on lexical selection. We explored this question in a follow-up study in which participants listened to similar sentence fragments while producing an upward or downward head movement with eyes being closed and producing suitable sentence endings with heads up versus down. We replicated the effects of sentence spatial properties on the spatial properties of produced nouns which are the focus of this article. In addition, we found an effect of head movement on the locations of produced nouns in this study which we interpret as evidence for the position that a substantial amount of experiential reactivation is needed to have an influence on lexical access (Vogt et al., 2022).

An additional factor which might have contributed to the absence of an effect of visual sentence movement is a lack of variability in spatial location of produced words as most words were rated as rather downwards related. Given the data from the head movement study where we also observed that produced words tended to be more downwards than upwards, we do not consider this lack of variability as the best explanation for the absence of the expected effect.

In contrast to the purely physical visual stimulation, the spatial context manipulation conveyed by the sentences carried more meaning. Indeed, the produced words were influenced by the spatial characteristics of the presented sentence fragments. For example, after reading a sentence like “You are at the sea and you see. . .” participants were more likely to say “a shell” than “a gull.” Crucially, both shells and gulls can be found at the sea. Furthermore, both words get assigned a comparable semantic similarity when using distributional measures of semantics as we did in our study, sea–shell: 0.40; sea–gull: 0.41; on a scale from 0 (*no similarity*) to 1 (*synonyms*). However, words additionally sharing the spatial location with the situation described by the sentences were more likely to be selected.

We take this as evidence that experiential knowledge not only affects the way word meaning is represented but also that it is activated during lexical-semantic planning stages, thereby influencing which words we choose. It has been shown by Ostarek and Vigliocco (2017) that identification of pictorial stimuli was facilitated when presented 250 ms after reading words which belong to the same event (e.g., reading “sky” and seeing “cloud”) when the image was presented in the same vertical location where it is typically seen which demonstrates the importance of events during perceptual simulation. Therefore, we deem it likely that in our experiments, participants simulate the scenes described by the sentences and that these simulations modulate conceptual and lexical processing. According to situation model theory, specifically the event-indexing model (Zwaan & Radvansky, 1998), space is an important meaning dimension when it comes to integrating different pieces of information given in the linguistic input. From this perspective, it is not surprising that participants produced words that share the spatial properties of the simulations they created when reading the previous linguistic input. In general, it seems that situation model theory (e.g., Zwaan, 2016) fits well with our results, assuming a division of labour between more symbolic and more grounded representations in discourse and thus providing a good explanation for the combined effects of semantic similarity and the more experientially grounded meaning dimension observed in this study.

To summarise, while many other factors regarding the selected content words may play a role during the lexical selection process, we want to highlight that experientially grounded meaning seems to be one important factor in language production. Also, we would like to point out that our results fit well with situation model theory.

### The relation between experientially grounded meaning and predictability

It might be argued that the produced words are all more or less predictable given the sentential context in the sense that most of them would probably not lead to processing difficulties when presented in a comprehension task and that it is therefore important to clarify whether the observed effect is carried by spatial location specifically or more generally by predictability. However, even when only examining the stimuli where participants showed most variability in answering, that is, at least on average every second participant produced a different word, we still obtained the main effect that spatial locations of the stimulus material predicted the spatial properties of the produced nouns.

We think that the notion of predictability with regard to lexical selection is empirically underspecified so far and that more research should be done to investigate which factors contribute to words being predicted in a given context. Our data show that experientially grounded meaning facets might be among those factors. In addition, statistical distributional properties of language might be important for predictability.

### The relation between experientially grounded meaning and traditional semantic measures

The experience-related manipulation of space employed here is embedded in meaningful contexts, but at the same time distinct from semantic context measures known to affect lexical-semantic processing during language production, such as semantic features, associations, thematic relations, or categories (Abdel Rahman & Melinger, 2019; McRae et al., 2005). To further examine the influence of the semantic contents of the presented sentences on the produced nouns, we included a distributional semantic similarity measure as a covariate in our analysis. We used cosine values computed from text corpora as they are an established measure in the field of semantics and therefore they provided a pragmatic way to yield similarity values for the large data set at hand. As the estimates of semantic similarity are based on huge language corpora, they pick up on the statistical linguistic regularities which we encounter in our daily life and are therefore very strong tools in modelling our linguistic behaviour. Even though distributional measures of semantics have hardly been incorporated into research on semantic processing in language production (Vinson et al., 2014), they are in our view perfectly suited for quantifying the semantic relationship between the nouns in the sentence fragments and the produced nouns. In Experiment 2, we found a more pronounced effect of spatial characteristics of the presented sentence on the spatial properties of the produced nouns for semantically related relative to unrelated pairs. More precisely, the closer the produced noun was to the content in the visually presented sentence, the stronger the impact of spatial location of the presented sentence fragment on the spatial location of the produced noun. Note that this interaction between similarity and sentence spatial location was only apparent in Experiment 2. This suggests that the presented article in the first experiment made it more difficult for the semantically most associated nouns to be produced. Indeed, the mean cosine value across all trials was lower in Experiment 1 (mean cosine value: 0.21) than in Experiment 2 (mean cosine value: 0.32), that is, produced nouns were overall less semantically related to the content of sentences in Experiment 1 than in Experiment 2. Thus, the design of Experiment 1, in which a determiner restricted the range of possible nouns, made it less likely that the produced nouns were chosen merely because their semantic association to the sentence context was strongest.

Crucially, however, we found that the spatial location values of the sentence nouns predicted the spatial properties of the produced nouns in Experiment 1. Moreover, in Experiment 2, the effect of sentence spatial location on spatial characteristics of the produced nouns was still existent in cases of minimal and high similarity relation. That is, even among the most loosely related cases, nouns were chosen which shared the spatial dimension on top. Therefore, the semantic similarity measure used here cannot entirely explain the relationship between the spatial characteristics of the sentence material and the produced nouns. Rather, similarity seems to work as a moderator, influencing the strength of the impact of spatial properties of the stimuli on the dependent variable. We conclude that meaning aspects as captured by the similarity measure and experientially grounded sensory meaning are closely entangled, but distinguishable, in line with theoretical accounts (Louwerse, 2018; Vigliocco et al., 2009).

Similar results have recently been obtained by Banks et al. (2021). Using a category production task, they were able to predict performance when taking both shared sensorimotor knowledge and linguistic proximity based on distributional knowledge into account. This goes in line with our interpretation that experiential and linguistic associations are both important, contributing separately to the responses we found.

## Conclusion

In this study, we show that lexical-semantic processes during language production are not influenced by physical spatial cues isolated from meaning. Instead, we provide evidence that lexical choices are influenced by experientially grounded sensory meaning of space—as conveyed by the verbal context—and that these choices are modulated by distributional properties of the linguistic context. This is in line with current hybrid theories of semantic memory, which treat sensorimotor aspects and usage-based distributional aspects of language as separate but interacting types of meaning (Carota et al., 2021; Davis & Yee, 2021; Vigliocco et al., 2009).

We propose that message planning for speaking does not only involve classic semantic meaning relations as categorical or associative links but may also include other aspects of meaning grounded in sensory, motor, or bodily experiences. Future research should study whether the impact of the meaning dimension of “location in space” is captured best as a reactivation of sensorimotor experiences, and thus constitutes evidence for experiential grounding in language production, or whether spatial locations are activated as part of propositional and amodal semantic features (Meteyard et al., 2012). However, based on the evidence reviewed in the “Introduction” and evidence for the activation of spatial–oculomotor regions in the brain during the processing of implicitly spatial nouns (Ostarek, 2018), we deem the meaning aspect of “locations in space” a strong candidate for experientially grounded meaning.

In the experimental task employed here, we investigated which words are chosen during lexical-semantic processing. Traditionally, most studies dealing with lexical access in language production manipulate the context of an utterance, whereas the to-be-produced word is pre-determined by the experimental setup (various picture naming tasks, e.g., cyclic blocking, picture-word interference, and continuous naming). Production tasks with a focus on semantics rarely allow for free lexical choices even though recent studies have moved in this direction (e.g., Fjaellingsdal et al., 2020). Here, participants were not entirely unrestricted in their lexical choices, but could freely select their utterances within non-constraining contexts, allowing us to investigate which factors shape the content of a produced message, rather than the duration of lexical processing. As is typical in everyday language use, our task also encompassed an interplay of comprehension and production (Indefrey & Levelt, 2004; Pickering & Garrod, 2013). Therefore, our experiments provide an important step towards a more complete understanding of one of the crucial elements of language production and we hope to spark interesting discussions and studies which will shed more light on the factors which contribute to answering the question why we choose certain words to express an intended meaning.

## Supplemental Material

sj-docx-1-qjp-10.1177_17470218221125425 – Supplemental material for Experience-driven meaning affects lexical choices during language production Click here for additional data file. Supplemental material, sj-docx-1-qjp-10.1177_17470218221125425 for Experience-driven meaning affects lexical choices during language production by Anne Vogt, Barbara Kaup and Rasha Abdel Rahman in Quarterly Journal of Experimental Psychology

## Notes

1 We also collected ratings for the spatial locations of the whole sentence fragments. Participants rated the spatial position of these sentences independently of how they might be completed. Entering these sentence spatial locations as predictors yielded the same effect with no effect for presentation direction and even stronger increases in slope for each level of spatial location from 1 (down) to 7 (up), (β = .38 [.27, .49], *t* = 6.78, *p* < .001).

2 Sample size for Experiment 2 was based on preliminary results of Experiment 1 hinging on spatial rating data for the complete set of produced nouns stemming from three subjects only. This analysis yielded a significant effect of presentation direction for cases with high semantic similarity between sentence noun and produced noun. Based on this finding, sample size for Experiment 2 was estimated (see preregistration). Even though the preliminary results turned out to be spurious after completing the spatial rating with 37 raters, we ran the second experiment with the originally planned sample size.

3 A 9-point Likert-type scale for prerating the experimental items for Experiment 2 was chosen as we assumed that a wider range would better pick up on the big real word differences in spatial locations. However, as suggested in several methodological papers, 7-point Likert-type scales seem to be yielding the best reliability (Cicchetti et al., 1985; Finn, 1972; Oaster, 1989; Ramsay, 1973); we again chose a 7-point Likert-type scale for the rating of the produced nouns as this also enables direct comparison of results from Experiments 1 and 2.

4 While 35 participants took part in Experiment 1 with 90 stimulus sentence, 72 participants took part in Experiment 2 with 60 stimulus sentences. Thereby, the total amount of potentially produced words which had to be rated already went up by ca. 25%. Furthermore, several of the raters in Experiment 1 gave us the feedback that they found it annoying to rate spatial locations of seemingly equivalent objects. Our decision to only let people rate one version of near-synonyms thereby served both the purpose of making raters more willing to cooperate and of reducing the amount of words to be rated and thereby reducing the time needed for doing the rating and/or the amount of raters. For Experiment 2, two experimenters decided together whether two produced words could be handled as synonyms for the rating and in case of doubt both versions were kept in the rating. For this decision, we always kept in mind whether two words would refer to the same type of object and whether the use of one word version versus the other could potentially have an impact on how far up or down in the world other people might perceive the word’s referents.

5 We deviated from the preregistered analysis plan of analysing the impact of semantic similarity in bins of 10% percentiles for two reasons. First, while cutting the whole set of similarities in percentile-bins would have permitted to run analyses with the same number of trials, it would have resulted in unequally spaced bins across the range of semantic similarity with the outer percentiles spanning a relatively large range of similarity values which is not informative with regard to the hypotheses. Second, splitting the whole set of similarities into 10 equally spaced ranges would have resulted in outer bins not containing enough trials to run statistical analyses. Therefore, we resorted to splitting up the whole range of obtained similarity values using five levels.
