# Supplementary material for: Experience-driven meaning affects lexical choices during language production
Source: Q J Exp Psychol (Hove). 2022 Oct 6;76(7):1561–84. doi: 10.1177/17470218221125425 (PMC10280667; doi:10.1177/17470218221125425)
Supplement: sj-docx-1-qjp-10.1177_17470218221125425 – Supplemental material for Experience-driven meaning affects lexical choices during language production [file sj-docx-1-qjp-10.1177_17470218221125425.docx]

Supplementary Material for:

**Experience-driven meaning affects lexical choices during language production**

Anne Vogt^1,2^, Barbara Kaup^3^ & Rasha Abdel Rahman^1,2^

1 Department of Psychology, Humboldt-Universität zu Berlin

2 Berlin School of Mind and Brain

3 University of Tübingen

Corresponding authors:

Anne Vogt, anne.vogt.1@hu-berlin.de, Rudower Chaussee 18, 12489 Berlin

Rasha Abdel Rahman, rasha.abdel.rahman@hu-berlin.de, Rudower Chaussee 18, 12489 Berlin

**Supplementary Material 1**

Table S1

*Experiment 1. LMM statistics for direction, sentence spatial location and cosine values serving as similarity measure computed with the semantic space de_wiki. Random effects for subjects were not included due to convergence errors.*

| Variable | *b* | *CI* | *t* | *p* |
| --- | --- | --- | --- | --- |
| Intercept | 3.57 | [3.49; 3.66] | 83.05 | <.001 |
| Direction  (Ascending-Descending) | 0.00 | [-0.09; 0.09] | -0.04 | .968 |
| Sentence Spatial Location | 0.20 | [0.11; 0.28] | 4.58 | <.001 |
| Semantic Similarity | ‑0.65 | [-1.06; -0.25] | ‑3.15 | .002 |
| Sentence Spatial Location :  Semantic Similarity | 0.20 | [-0.21; 0.62] | 0.97 | .335 |

*Note.* The effect of spatial similarity cannot be interpreted meaningfully as the predicted values were spatial characteristics of the produced nouns. See Experiment 2, for discussion of a different impact of similarity on the predicted values

Table S2

*Experiment 2. LMM statistics for direction, sentence spatial location and cosine values serving as similarity measure computed with the semantic space de_wiki*

| Variable | *b* | *CI* | *t* | *p* |
| --- | --- | --- | --- | --- |
| Intercept | 3.72 | [3.61; 3.83] | 66.80 | <.001 |
| Direction  (Central-Descending) | -0.06 | [-0.15; 0.03] | -1.36 | .175 |
| Direction  (Ascending-Central) | -0.09 | [-0.18; 0.00] | -2.04 | .041 |
| Sentence Spatial Location | 0.30 | [0.21; 0.39] | 6.40 | <.001 |
| Semantic Similarity | -0.23 | [-0.55; 0.09] | -1.39 | 0.165 |
| Sentence Spatial Location :  Semantic Similarity | 0.90 | [0.63; 1.18] | 6.39 | <.001 |

Table S3

*Predictability Experiment 2. Table containing the most frequently produced noun for each stimulus sentence together with its cloze value demonstrating that predictability as indicated by cloze values is not a likely source of the effects we found with cloze values being generally low. The rightmost column contains the total number of different concepts produced for each stimulus sentence.*

| stimulus number | sentence noun | most frequently produced sentence ending | absolute count of the most frequently produced noun | cloze value (relative proportion of the most frequently produced noun) | number of different concepts |
| --- | --- | --- | --- | --- | --- |
| 1 | Meer | ein Strand | 9 | 0.125 | 34 |
| 2 | Pool | eine Luftmatratze | 9 | 0.125 | 34 |
|  |  | Wasser | 9 | 0.125 |  |
| 3 | Kanal | ein Boot | 14 | 0.194 | 37 |
| 4 | Fluss | ein Fisch | 9 | 0.125 | 37 |
| 5 | See | ein Boot | 8 | 0.111 | 40 |
| 6 | Teich | ein Frosch | 14 | 0.194 | 29 |
| 7 | Bach | ein Fisch | 21 | 0.292 | 28 |
| 8 | Feld | ein Hase | 8 | 0.111 | 47 |
| 9 | Ufer | ein Boot | 8 | 0.111 | 42 |
| 10 | Strand | Sand | 8 | 0.111 | 30 |
| 11 | Wiese | eine Blume | 15 | 0.208 | 38 |
| 12 | Straße | ein Auto | 23 | 0.319 | 28 |
| 13 | Kreuzung | eine Ampel | 26 | 0.361 | 17 |
| 14 | Lichtung | ein Reh | 16 | 0.222 | 35 |
| 16 | Garten | eine Blume | 15 | 0.208 | 45 |
| 17 | Zelt | ein Schlafsack | 8 | 0.111 | 36 |
| 18 | Sofa | ein Kissen | 15 | 0.208 | 22 |
| 19 | Freibad | ein Sprungbrett | 9 | 0.125 | 31 |
| 20 | Haltestelle | ein Bus | 31 | 0.431 | 18 |
| 21 | Weide | eine Kuh | 21 | 0.292 | 21 |
| 22 | Baustelle | ein Bagger | 14 | 0.194 | 30 |
| 23 | Bahnhof | ein Zug | 31 | 0.431 | 26 |
| 24 | Zug | ein Gleis | 5 | 0.069 | 26 |
| 25 | Garage | ein Auto | 29 | 0.403 | 23 |
| 26 | Eingang | eine Tür | 22 | 0.306 | 22 |
| 27 | Terrasse | die Sonne | 5 | 0.069 | 40 |
|  |  | ein Stuhl | 5 | 0.069 |  |
|  |  | ein Vogel | 5 | 0.069 |  |
|  |  | eine Blume | 5 | 0.069 |  |
| 28 | Innenhof | ein Fahrrad | 8 | 0.111 | 41 |
| 29 | Park | ein Hund | 15 | 0.208 | 32 |
| 30 | Küche | ein Kühlschrank | 10 | 0.139 | 38 |
| 31 | Bus | eine Haltestelle | 7 | 0.097 | 33 |
| 32 | Veranda | ein Garten | 7 | 0.097 | 36 |
| 33 | Ofen | ein Feuer | 10 | 0.139 | 31 |
|  |  | ein Kuchen | 10 | 0.139 |  |
| 34 | Badezimmer | ein Spiegel | 12 | 0.167 | 18 |
| 35 | Straßenbahn | ein Bus | 5 | 0.069 | 27 |
| 36 | Café | ein Tisch | 13 | 0.181 | 20 |
| 37 | Museum | ein Gemälde | 17 | 0.236 | 25 |
| 38 | Zaun | ein Hund | 5 | 0.069 | 41 |
|  |  | ein Vogel | 5 | 0.069 |  |
| 39 | Schuppen | eine Schaufel | 19 | 0.264 | 30 |
| 40 | Turnhalle | ein Ball | 14 | 0.194 | 29 |
| 41 | Schule | eine Schultafel | 17 | 0.236 | 28 |
| 42 | Haustür | ein Schlüssel | 9 | 0.125 | 27 |
| 43 | Dschungel | ein Affe | 14 | 0.194 | 22 |
| 44 | Schaufenster | eine Schaufensterpuppe | 29 | 0.403 | 20 |
| 45 | Tisch | ein Teller | 12 | 0.167 | 30 |
| 46 | Wald | ein Reh | 12 | 0.167 | 35 |
| 47 | Haus | eine Tür | 13 | 0.181 | 38 |
| 48 | Scheune | ein Pferd | 13 | 0.181 | 26 |
| 49 | Garderobe | eine Jacke | 25 | 0.347 | 20 |
| 50 | Kaufhaus | eine Kasse | 6 | 0.083 | 41 |
| 51 | Brücke | Wasser | 10 | 0.139 | 32 |
| 52 | Stadion | ein Tor | 8 | 0.111 | 21 |
| 53 | Baum | ein Vogel | 12 | 0.167 | 29 |
| 54 | Fenster | ein Vogel | 7 | 0.097 | 35 |
| 55 | Straßenlaterne | Licht | 14 | 0.194 | 36 |
| 56 | Balkon | eine Blume | 10 | 0.139 | 39 |
| 57 | Leuchtturm | das Meer | 21 | 0.292 | 26 |
| 58 | Gebirge | Berge | 10 | 0.139 | 34 |
| 59 | Baumhaus | ein Vogel | 5 | 0.069 | 43 |
| 60 | Aussichtspunkt | der Horizont | 5 | 0.069 | 37 |

**Supplementary Material 2 A**

| Item No. | Position 1 | Position 2 | Position 3 | Position 4 | Position 5 | Spatial Prerating  Sentence | Spatial Prerating Noun |
| --- | --- | --- | --- | --- | --- | --- | --- |
| 1 | Du | gehst | zu | der | Bushaltestelle | 4,22 | 3,56 |
|  | *You* | *walk* | *to* | *the* | *bus stop* |  |  |
| 2 | Du | radelst | über | das | Kornfeld | 4,11 | 3,56 |
|  | *You* | *cycle* | *across* | *the* | *corn field* |  |  |
| 3 | Du | fährst | durch | die | Innenstadt | 4,11 | 4,14 |
|  | *You* | *drive* | *through* | *the* | *city* |  |  |
| 4 | Du | schlurfst | über | den | Gehweg | 2,67 | 1,89 |
|  | *You* | *shuffle* | *across* | *the* | *pavement* |  |  |
| 5 | Du | balancierst | auf | dem | Spielplatz | 4,11 | 2,89 |
|  | *You* | *balance on the beam* | *at* | *the* | *playground* |  |  |
| 6 | Du | wandelst | über | die | Promenade | 4,33 | 3,11 |
|  | *You* | *stroll* | *along* | *the* | *promenade* |  |  |
| 7 | Du | paddelst | über | den | See | 4,11 | 2,78 |
|  | *You* | *paddle* | *across* | *the* | *lake* |  |  |
| 8 | Du | spazierst | über | das | Feld | 4,33 | 2,44 |
|  | *You* | *walk* | *across* | *the* | *field* |  |  |
| 9 | Du | schleichst | durch | den | Garten | 3,89 | 3,13 |
|  | *You* | *sneak* | *through* | *the* | *garden* |  |  |
| 10 | Du | bummelst | durch | die | Fußgängerzone | 4,11 | 3,13 |
|  | *You* | *stroll* | *through* | *the* | *pedestrian zone* |  |  |
| 11 | Du | sprintest | über | das | Fußballfeld | 3,78 | 1,89 |
|  | *You* | *sprint* | *across* | *the* | *pitch* |  |  |
| 12 | Du | läufst | über | den | Strand | 2,78 | 2,67 |
|  | *You* | *walk* | *on* | *the* | *beach* |  |  |
| 13 | Du | streifst | durch | das | Gebüsch | 2,67 | 3,67 |
|  | *You* | *roam* | *around* | *the* | *shrubbery* |  |  |
| 14 | Du | wanderst | durch | den | Wald | 3,67 | 5,25 |
|  | *You* | *hike* | *through* | *the* | *forest* |  |  |
| 15 | Du | rennst | über | den | Sportplatz | 4,00 | 2,25 |
|  | *You* | *run* | *across* | *the* | *sports field* |  |  |
| 16 | Du | springst | über | den | Baumstamm | 3,33 | 3,33 |
|  | *You* | *jump* | *over* | *the* | *tree trunk* |  |  |
| 17 | Du | schwimmst | durch | den | Fluss | 3,33 | 2,44 |
|  | *You* | *swim* | *through* | *the* | *river* |  |  |
| 18 | Du | schlenderst | durch | den | Park | 4,11 | 3,25 |
|  | *You* | *stroll* | *through* | *the* | *park* |  |  |
| 19 | Du | flanierst | durch | die | Gassen | 4,00 | 2,67 |
|  | *You* | *amble* | *through* | *the* | *streets* |  |  |
| 20 | Du | joggst | durch | die | Grünanlage | 3,44 | 3,13 |
|  | *You* | *jog* | *through* | *the* | *recreation area* |  |  |
| 21 | Du | hüpfst | über | die | Terrasse | 3,67 | 2,50 |
|  | *You* | *hop* | *across* | *the* | *terrace* |  |  |
| 22 | Du | gehst | zu | der | Weide | 3,67 | 2,89 |
|  | *You* | *go* | *to* | *the* | *meadow* |  |  |
| 23 | Du | radelst | über | den | Feldweg | 4,00 | 2,33 |
|  | *You* | *cycle* | *across* | *the* | *dirt road* |  |  |
| 24 | Du | fährst | auf | der | Autobahn | 3,89 | 2,33 |
|  | *You* | *drive* | *on* | *the* | *motor way* |  |  |
| 25 | Du | schlurfst | über | das | Kopfsteinpflaster | 2,67 | 1,44 |
|  | *You* | *shuffle* | *over* | *the* | *cobblestone* |  |  |
| 26 | Du | balancierst | auf | dem | Schulhof | 3,44 | 3,11 |
|  | *You* | *balance on a beam* | *in* | *the* | *schoolyard* |  |  |
| 27 | Du | wandelst | auf | die | Panoramapfad | 4,67 | 4,00 |
|  | *You* | *stroll* | *along* | *the* | *panorama path* |  |  |
| 28 | Du | paddelst | durch | den | Kanal | 3,33 | 2,00 |
|  | *You* | *paddle* | *across* | *the* | *canal* |  |  |
| 29 | Du | spazierst | entlang | der | Küste | 3,33 | 3,63 |
|  | *You* | *stroll* | *along* | *the* | *coastline* |  |  |
| 30 | Du | schleichst | durch | den | Hafen | 4,44 | 2,86 |
|  | *You* | *sneak* | *through* | *the* | *harbour* |  |  |
| 31 | Du | bummelst | durch | den | Schlosspark | 4,56 | 3,57 |
|  | *You* | *stroll* | *through* | *the* | *palace grounds* |  |  |
| 32 | Du | sprintest | auf | der | Rennbahn | 3,67 | 2,00 |
|  | *You* | *sprint* | *on* | *the* | *racetrack* |  |  |
| 33 | Du | läufst | zu | der | Schule | 3,89 | 4,00 |
|  | *You* | *walk* | *to* | *the* | *school* |  |  |
| 34 | Du | streifst | durch | die | Straßen | 4,11 | 2,00 |
|  | *You* | *roam* | *through* | *the* | *streets* |  |  |
| 35 | Du | wanderst | über | die | Bergalm | 5,22 | 6,29 |
|  | *You* | *hike* | *across* | *the* | *mountain pastures* |  |  |
| 36 | Du | rennst | über | den | Acker | 4,00 | 2,56 |
|  | *You* | *run* | *across* | *the* | *field* |  |  |
| 37 | Du | springst | über | den | Rasen | 4,44 | 2,11 |
|  | *You* | *jump* | *across* | *the* | *lawn* |  |  |
| 38 | Du | schwimmst | in | einem | Weiher | 3,33 | 2,22 |
|  | *You* | *swim* | *in* | *the* | *pond* |  |  |
| 39 | Du | schlenderst | über | das | Spielfeld | 4,00 | 2,22 |
|  | *You* | *stroll* | *through* | *the* | *playing field* |  |  |
| 40 | Du | flanierst | über | den | Markt | 4,00 | 3,63 |
|  | *You* | *amble* | *across* | *the* | *market* |  |  |
| 41 | Du | joggst | entlang | des | Flussufers | 3,33 | 2,11 |
|  | *You* | *jog* | *along* | *the* | *riverbank* |  |  |
| 42 | Du | hüpfst | über | das | Moos | 2,89 | 1,77 |
|  | *You* | *hop* | *over* | *the* | *moss* |  |  |
| 43 | Du | gehst | über | die | Pferdekoppel | 4,00 | 3,11 |
|  | *You* | *go* | *across* | *the* | *paddock* |  |  |
| 44 | Du | radelst | auf | dem | Waldweg | 3,89 | 2,89 |
|  | *You* | *cycle* | *on* | *the* | *forest path* |  |  |
| 45 | Du | fährst | über | die | Kreuzung | 3,78 | 3,00 |
|  | *You* | *drive* | *over* | *the* | *crossing* |  |  |
| 46 | Du | schlurfst | zu | dem | Gartentor | 2,89 | 3,56 |
|  | *You* | *shuffle* | *to* | *the* | *garden gate* |  |  |
| 47 | Du | balancierst | auf | dem | Geländer | 3,33 | 3,67 |
|  | *You* | *balance* | *on* | *the* | *balustrade* |  |  |
| 48 | Du | wandelst | durch | den | Klostergarten | 3,56 | 3,25 |
|  | *You* | *walk* | *through* | *the* | *monastery garden* |  |  |
| 49 | Du | paddelst | um | die | Insel | 4,00 | 4,00 |
|  | *You* | *paddle* | *around* | *the* | *island* |  |  |
| 50 | Du | spazierst | durch | die | Altstadt | 4,67 | 3,71 |
|  | *You* | *stroll* | *through* | *the* | *old town* |  |  |
| 51 | Du | schleichst | durch | das | Unterholz | 2,11 | 2,33 |
|  | *You* | *sneak* | *through* | *the* | *brushwood* |  |  |
| 52 | Du | bummelst | auf | dem | Schulweg | 3,56 | 2,78 |
|  | *You* | *wander* | *on* | *the* | *way to school* |  |  |
| 53 | Du | sprintest | zu | der | Brombeerhecke | 3,67 | 3,89 |
|  | *You* | *sprint* | *to* | *the* | *blackberry bush* |  |  |
| 54 | Du | läufst | über | den | Friedhof | 2,78 | 3,00 |
|  | *You* | *walk* | *through* | *the* | *cemetery* |  |  |
| 55 | Du | streifst | durch | die | Landschaft | 4,33 | 3,50 |
|  | *You* | *roam* | *through* | *the* | *countryside* |  |  |
| 56 | Du | wanderst | entlang | des | Bachs | 3,11 | 2,22 |
|  | *You* | *hike* | *along* | *the* | *creek* |  |  |
| 57 | Du | rennst | durch | das | Stadion | 4,67 | 4,11 |
|  | *You* | *run* | *through* | *the* | *stadium* |  |  |
| 58 | Du | springst | über | das | Hindernis | 3,11 | 3,89 |
|  | *You* | *jump* | *over* | *the* | *barrier* |  |  |
| 59 | Du | schwimmst | durch | den | Bodensee | 3,22 | 1,75 |
|  | *You* | *swim* | *in* |  | *Lake Constance* |  |  |
| 60 | Du | schlenderst | über | den | Markusplatz | 4,56 | 3,17 |
|  | *You* | *stroll* | *across* |  | *Saint Marcs Square* |  |  |
| 61 | Du | flanierst | durch | die | Landesgartenschau | 3,00 | 3,80 |
|  | *You* | *amble* | *through* | *the* | *garden festival* |  |  |
| 62 | Du | joggst | in | einem | Naturschutzgebiet | 3,44 | 3,40 |
|  | *You* | *jog* | *in* | *a* | *wildlife sanctuary* |  |  |
| 63 | Du | hüpfst | über | die | Steinplatten | 3,89 | 3,13 |
|  | *You* | *hop* | *over* | *the* | *slabs* |  |  |
| 64 | Du | wartest | auf | den | Zug | 4,25 | 4,67 |
|  | *You* | *wait* | *for* | *the* | *train* |  |  |
| 65 | Du | lehnst | an | einem | Fenster | 4,78 | 4,44 |
|  | *You* | *lean* | *against* | *a* | *window* |  |  |
| 66 | Du | sitzt | auf | einer | Bank | 4,11 | 3,44 |
|  | *You* | *sit* | *on* | *a* | *bench* |  |  |
| 67 | Du | hockst | auf | einem | Felsen | 3,78 | 5,00 |
|  | *You* | *squat* | *on* | *a* | *rock* |  |  |
| 68 | Du | stehst | an | einem | Seeufer | 4,11 | 2,44 |
|  | *You* | *stand* | *on* | *a* | *lakeshore* |  |  |
| 69 | Du | liegst | auf | der | Wiese | 4,67 | 2,00 |
|  | *You* | *lie* | *on* | *the* | *lawn* |  |  |
| 70 | Du | rastest | auf | einer | Parkbank | 3,44 | 3,33 |
|  | *You* | *rest* | *on* | *a* | *park bench* |  |  |
| 71 | Du | thronst | auf | einem | Baumstumpf | 4,44 | 2,22 |
|  | *You* | *sit* | *on* | *a* | *tree stump* |  |  |
| 72 | Du | kniest | zwischen | den | Erdbeeren | 1,33 | 2,00 |
|  | *You* | *kneel* | *among* | *the* | *strawberries* |  |  |
| 73 | Du | wartest | auf | den | Bus | 4,13 | 4,22 |
|  | *You* | *wait* | *for* | *the* | *bus* |  |  |
| 74 | Du | lehnst | an | einer | Wand | 3,67 | 4,63 |
|  | *You* | *lean* | *against* | *a* | *wall* |  |  |
| 75 | Du | sitzt | in | der | Hollywoodschaukel | 4,11 | 3,78 |
|  | *You* | *sit* | *in* | *the* | *canopy swing* |  |  |
| 76 | Du | hockst | neben | dem | Misthaufen | 3,22 | 2,89 |
|  | *You* | *squat* | *next to* | *the* | *dungpile* |  |  |
| 77 | Du | stehst | vor | einem | Kaufhaus | 3,89 | 4,50 |
|  | *You* | *stand* | *in front of* | *a* | *mall* |  |  |
| 78 | Du | liegst | auf | einem | Handtuch | 4,67 | 3,14 |
|  | *You* | *lie* | *on* | *a* | *towel* |  |  |
| 79 | Du | rastest | bei | einem | Wegweiser | 4,11 | 4,67 |
|  | *You* | *rest* | *beneath* | *a* | *signpost* |  |  |
| 80 | Du | thronst | auf | der | Rutsche | 3,89 | 4,44 |
|  | *You* | *sit* | *on* | *the* | *slide* |  |  |
| 81 | Du | kniest | auf | dem | Bürgersteig | 1,89 | 2,11 |
|  | *You* | *kneel* | *on* | *the* | *pavement* |  |  |
| 82 | Du | wartest | vor | einem | Café | 3,67 | 4,00 |
|  | *You* | *wait* | *in front of* | *a* | *café* |  |  |
| 83 | Du | lehnst | an | einer | Straßenlaterne | 4,00 | 5,89 |
|  | *You* | *lean* | *against* | *a* | *street lantern* |  |  |
| 84 | Du | sitzt | auf | einer | Wippe | 3,75 | 4,33 |
|  | *You* | *sit* | *on* | *a* | *seesaw* |  |  |
| 85 | Du | hockst | in | einem | Sandkasten | 1,78 | 1,89 |
|  | *You* | *squat* | *in* | *a* | *sandpit* |  |  |
| 86 | Du | stehst | an | einer | Ampel | 4,33 | 5,33 |
|  | *You* | *stand* | *at* | *a* | *traffic light* |  |  |
| 87 | Du | liegst | auf | der | Picknickdecke | 4,00 | 1,78 |
|  | *You* | *lie* | *on* | *a* | *picnic blanket* |  |  |
| 88 | Du | rastest | auf | einer | Lichtung | 3,89 | 3,38 |
|  | *You* | *rest* | *on* | *a* | *clearing* |  |  |
| 89 | Du | thronst | auf | einem | Steinbrocken | 4,56 | 3,50 |
|  | *You* | *sit* | *on* | *a* | *boulder* |  |  |
| 90 | Du | kniest | neben | dem | Trampolin | 3,44 | 4,89 |
|  | *You* | *kneel* | *next to* | *the* | *trampoline* |  |  |

*Note.* All the sentences continued with ‘*und du siehst/ erblickst / entdeckst ein/e/n …*’ (English ‘and you see / spot / discover a …’). The verb describing perception at the 7th position was fixed for each sentence, stimulus sentence 1 contained the verb ‘see’, stimulus sentence 2 contained the verb ‘spot’, stimulus sentence 3 contained the verb ‘discover’, stimulus sentence 4 contained the verb ‘see’ and so on. The article at the 8th position was counterbalanced across experimental lists

**Supplementary Material 2 B**

*Stimuli for Experiment 2 with original German sentences and English translation up to 4th sentence position and spatial prerating values.*

| Item No. | Position 1 | Position 2 | Position 3 | Position 4 | Spatial Prerating Noun | Triplet No. |
| --- | --- | --- | --- | --- | --- | --- |
| 1 | Du | bist | am | Meer | 2,60 | 1 |
|  | *You* | *are* | *at the* | *sea* |  |  |
| 2 | Du | bist | am | Pool | 2,80 | 1 |
|  | *You* | *are* | *at the* | *pool* |  |  |
| 3 | Du | stehst | am | Kanal | 2,40 | 1 |
|  | *You* | *stand* | *at the* | *canal* |  |  |
| 4 | Du | läufst | zum | Fluss | 2,80 | 2 |
|  | *You* | *walk* | *to the* | *river* |  |  |
| 5 | Du | stehst | am | See | 2,93 | 2 |
|  | *You* | *stand* | *at the* | *lakeshore* |  |  |
| 6 | Du | gehst | zum | Teich | 2,80 | 2 |
|  | *You* | *go* | *to the* | *pond* |  |  |
| 7 | Du | läufst | zum | Bach | 3,00 | 3 |
|  | *You* | *walk* | *to the* | *creek* |  |  |
| 8 | Du | läufst | zum | Feld | 3,07 | 3 |
|  | *You* | *walk* | *to the* | *field* |  |  |
| 9 | Du | stehst | am | Ufer | 3,15 | 3 |
|  | *You* | *stand* | *at the* | *riverbank* |  |  |
| 10 | Du | bist | am | Strand | 3,21 | 4 |
|  | *You* | *are* | *at the* | *beach* |  |  |
| 11 | Du | betrittst | die | Wiese | 3,29 | 4 |
|  | *You* | *enter* | *the* | *meadow* |  |  |
| 12 | Du | gehst | zur | Straße | 3,40 | 4 |
|  | *You* | *go* | *to the* | *street* |  |  |
| 13 | Du | läufst | zur | Kreuzung | 3,71 | 5 |
|  | *You* | *walk* | *to the* | *crossing* |  |  |
| 14 | Du | läufst | zur | Lichtung | 3,71 | 5 |
|  | *You* | *walk* | *to the* | *clearing* |  |  |
| 15 | Du | stehst | am | Hafen | 3,71 | 5 |
|  | *You* | *stand* | *at the* | *harbour* |  |  |
| 16 | Du | bist | im | Garten | 3,85 | 6 |
|  | *You* | *are* | *in the* | *garden* |  |  |
| 17 | Du | gehst | zum | Zelt | 3,87 | 6 |
|  | *You* | *go* | *to the* | *tent* |  |  |
| 18 | Du | gehst | zum | Sofa | 3,87 | 6 |
|  | *You* | *go* | *to the* | *sofa* |  |  |
| 19 | Du | bist | im | Freibad | 4 | 7 |
|  | *You* | *are* | *at the* | *pool* |  |  |
| 20 | Du | gehst | zur | Haltestelle | 3,92 | 7 |
|  | *You* | *go* | *to the* | *bus stop* |  |  |
| 21 | Du | betrittst | die | Weide | 3,93 | 7 |
|  | *You* | *walk* | *across the* | *meadow* |  |  |
| 22 | Du | betrittst | die | Baustelle | 4,00 | 8 |
|  | *You* | *enter* | *the* | *construction site* |  |  |
| 23 | Du | stehst | am | Bahnhof | 4,00 | 8 |
|  | *You* | *stand* | *at the* | *train station* |  |  |
| 24 | Du | gehst | zum | Zug | 4,00 | 8 |
|  | *You* | *go* | *to the* | *train* |  |  |
| 25 | Du | läufst | zur | Garage | 4,14 | 9 |
|  | *You* | *walk* | *to the* | *garage* |  |  |
| 26 | Du | stehst | am | Eingang | 4,20 | 9 |
|  | *You* | *stand* | *at the* | *entrance* |  |  |
| 27 | Du | gehst | zur | Terrasse | 4,07 | 9 |
|  | *You* | *go* | *to the* | terrace |  |  |
| 28 | Du | betrittst | den | Innenhof | 4,21 | 10 |
|  | *You* | *enter* | *the* | *courtyard* |  |  |
| 29 | Du | bist | im | Park | 4,23 | 10 |
|  | *You* | *are* | *in the* | *park* |  |  |
| 30 | Du | betrittst | die | Küche | 4,21 | 10 |
|  | *You* | *enter* | *the* | *kitchen* |  |  |
| 31 | Du | läufst | zum | Bus | 4,29 | 11 |
|  | *You* | *walk* | *to the* | *bus* |  |  |
| 32 | Du | betrittst | die | Veranda | 4,36 | 11 |
|  | *You* | *enter* | *the* | *veranda* |  |  |
| 33 | Du | stehst | am | Ofen | 4,29 | 11 |
|  | *You* | *stand* | *by the* | *stove* |  |  |
| 34 | Du | betrittst | das | Badezimmer | 4,43 | 12 |
|  | *You* | *enter* | *the* | *bathroom* |  |  |
| 35 | Du | läufst | zur | Straßenbahn | 4,40 | 12 |
|  | *You* | *walk* | *to the* | *tram* |  |  |
| 36 | Du | betrittst | das | Café | 4,43 | 12 |
|  | *You* | *enter* | *the* | *café* |  |  |
| 37 | Du | bist | im | Museum | 4,57 | 13 |
|  | *You* | *are* | *at the* | *museum* |  |  |
| 38 | Du | stehst | am | Zaun | 4,50 | 13 |
|  | *You* | *stand* | *by the* | *fence* |  |  |
| 39 | Du | gehst | zum | Schuppen | 4,47 | 13 |
|  | *You* | *go* | *to the* | *shed* |  |  |
| 40 | Du | betrittst | die | Turnhalle | 4,64 | 14 |
|  | *You* | *enter* | *the* | *gym* |  |  |
| 41 | Du | betrittst | die | Schule | 4,57 | 14 |
|  | *You* | *enter* | *the* | *school* |  |  |
| 42 | Du | gehst | zur | Haustür | 4,60 | 14 |
|  | *You* | *go* | *to the* | *entrance door* |  |  |
| 43 | Du | bist | im | Dschungel | 4,75 | 15 |
|  | *You* | *are* | *in the* | *jungle* |  |  |
| 44 | Du | stehst | am | Schaufenster | 4,71 | 15 |
|  | *You* | *stand* | *by the* | *shop window* |  |  |
| 45 | Du | gehst | zum | Tisch | 4,73 | 15 |
|  | *You* | *go* | *to the* | *table* |  |  |
| 46 | Du | bist | im | Wald | 4,86 | 16 |
|  | *You* | *are* | *in the* | *forest* |  |  |
| 47 | Du | läufst | zum | Haus | 5,07 | 16 |
|  | *You* | *walk* | *to the* | *house* |  |  |
| 48 | Du | gehst | zur | Scheune | 4,93 | 16 |
|  | *You* | *go* | *to the* | *barn* |  |  |
| 49 | Du | läufst | zur | Garderobe | 5,33 | 17 |
|  | *You* | walk | *to the* | *cloak room* |  |  |
| 50 | Du | betrittst | das | Kaufhaus | 5,50 | 17 |
|  | *You* | *enter* | *the* | *department store* |  |  |
| 51 | Du | gehst | zur | Brücke | 5,50 | 17 |
|  | *You* | *go* | *to the* | *bridge* |  |  |
| 52 | Du | bist | im | Stadion | 5,71 | 18 |
|  | *You* | *are* | *in the* | *stadium* |  |  |
| 53 | Du | läufst | zum | Baum | 5,93 | 18 |
|  | *You* | *walk* | *to the* | *tree* |  |  |
| 54 | Du | stehst | am | Fenster | 5,87 | 18 |
|  | *You* | *stand* | *by the* | *window* |  |  |
| 55 | Du | läufst | zur | Straßenlaterne | 6,60 | 19 |
|  | *You* | *walk* | *to the* | *street light* |  |  |
| 56 | Du | betrittst | den | Balkon | 6,57 | 19 |
|  | *You* | *enter* | *the* | *balcony* |  |  |
| 57 | Du | stehst | am | Leuchtturm | 6,60 | 19 |
|  | *You* | *stand* | *by the* | *light house* |  |  |
| 58 | Du | bist | im | Gebirge | 7,29 | 20 |
|  | *You* | *are* | *in the* | *mountains* |  |  |
| 59 | Du | bist | im | Baumhaus | 7,60 | 20 |
|  | *You* | *are* | *in the* | *tree house* |  |  |
| 60 | Du | stehst | am | Aussichtspunkt | 7,73 | 20 |
|  | *You* | *stand* | *on a* | *vantage point* |  |  |
